# A Delphi Study to Strengthen Research-Methods Training in Undergraduate Psychology Programs

**DOI:** 10.1177/25152459231213808

**Published:** 2024-01-29

**Authors:** Robert T. Thibault, Deborah Bailey-Rodriguez, James E. Bartlett, Paul Blazey, Robin J. Green, Madeleine Pownall, Marcus R. Munafò

**Affiliations:** 1 Meta-Research Innovation Center at Stanford, Stanford University, Stanford, California; 2 School of Psychological Science, University of Bristol, Bristol, England; 3 Psychology Department, Faculty of Science & Technology, Middlesex University, London, England; 4 School of Psychology and Neuroscience, University of Glasgow, Glasgow, Scotland; 5 Faculty of Medicine, Department of Family Practice, University of British Columbia, Vancouver, British Columbia, Canada; 6 School of Psychology and Vision Sciences, University of Leicester, Leicester, England; 7 School of Psychology, University of Leeds, Leeds, England; 8 MRC Integrative Epidemiology Unit at the University of Bristol, Bristol, England

**Keywords:** Delphi, psychology education, research methods, consensus, British Psychological Society, accreditation standards, undergraduate, replication, statistics, qualitative methods, research design

## Abstract

Psychology programs often emphasize inferential statistical tests over a solid understanding of data and research design. This imbalance may leave graduates underequipped to effectively interpret research and employ data to answer questions. We conducted a two-round modified Delphi to identify the research-methods skills that the UK psychology community deems essential for undergraduates to learn. Participants included 103 research-methods instructors, academics, students, and nonacademic psychologists. Of 78 items included in the consensus process, 34 reached consensus. We coupled these results with a qualitative analysis of 707 open-ended text responses to develop nine recommendations for organizations that accredit undergraduate psychology programs—such as the British Psychological Society. We recommend that accreditation standards emphasize (1) data skills, (2) research design, (3) descriptive statistics, (4) critical analysis, (5) qualitative methods, and (6) both parameter estimation and significance testing; as well as (7) give precedence to foundational skills, (8) promote transferable skills, and (9) create space in curricula to enable these recommendations. Our data and findings can inform modernized accreditation standards to include clearly defined, assessable, and widely encouraged skills that foster a competent graduate body for the contemporary world.

For graduates from psychology programs to thrive, they must become effective thinkers in a data-laden world. Research-methods education in psychology programs, however, often emphasizes inferential statistical tests over a deep understanding of data and research design (TARG Meta-Research Group, 2022), which could lead to the problematic use and interpretation of statistics. Moreover, open research practices are not yet embedded in many curricula, and qualitative research methods often remain underemphasized. In the UK, the British Psychological Society (BPS; 2019) sets the requirements for the vast majority of undergraduate psychology programs through their accreditation standards. Here, we conducted a consensus process, open to the UK psychology community, with the aim to strengthen the research-methods section of the BPS accreditation standards.

In response to the “replication crisis,” psychology researchers have increasingly adopted open science practices and considered statistical power, sample size, and the use of estimation (Cumming & Calin-Jageman, 2016). These advances, however, are not yet well reflected in psychology curricula. At least five studies have assessed the content of university-level psychology programs in the United States and generally concluded that there have been few updates to the curricula over the past 2 or 3 decades (Aiken et al., 1990, 2008; Anglin & Edlund, 2020; Friedrich et al., 2000, 2018). A similar study in the UK found that only 19% of universities had publicly available syllabi describing the content taught in each of their statistics modules in undergraduate psychology (TARG Meta-Research Group, 2022). Although these syllabi rarely contained a lesson-by-lesson breakdown, most mentioned specific inferential statistical tests (e.g., analyses of variance); about half mentioned probability and randomness, effect size, and statistical power; and few mentioned concepts such as confidence intervals, multiple comparisons, meta-analysis, replication, Bayesian statistics, frequentist statistics, and practical significance. Another study surveyed psychology students and instructors in the UK and found that few courses teach alternatives to null hypothesis significance testing (NHST) and that students’ anxiety around mathematics and statistics hold them back (Field, 2014). Note that a British Academy report highlighted this issue in stating that “a co-ordinated and continuous effort at improving quantitative skills across all phases of education and employment, in all four nations of the UK, is therefore now urgently needed” (The British Academy, 2015).

Qualitative research skills have been part of the BPS accreditation standards since 2004. However, there is limited research on how they are taught, and instructors may need additional training and resources to effectively teach qualitative methods (Gibson & Sullivan, 2018; Hugh-Jones et al., 2012; Wiggins et al., 2016). Some people also view qualitative research methods as the alternative and “lesser” approach to quantitative approaches, thus affecting how they are taught (Gibson & Sullivan, 2018; Hugh-Jones et al., 2012). Taken together, the time is ripe to modernize the teaching of quantitative and qualitative research methods in psychology programs.

We conducted the present consensus process with the goal of informing an updated version of the BPS *Standards for the Accreditation of Undergraduate, Conversion and Integrated Masters Programmes in Psychology*, specifically Section 2.1.4g of this document (BPS, 2019). Similar consensus processes have been used to develop standards in more than 200 medical education programs (Humphrey-Murto et al., 2017). The updated accreditation standards could contain specific actionable items for UK psychology research-methods curricula that reflect the need for data skills in the modern world and are adapted to the evolving educational landscape. Our results also provide a foundation for organizations beyond the BPS who seek to modernize research-methods education in undergraduate psychology programs.

## Study Objective

Our objective was to achieve consensus regarding the accreditation standards for research-methods education in undergraduate psychology programs in the UK, specifically, to provide the BPS with information to update their accreditation standards for research-methods education. (For contextual information regarding BPS accreditation and UK undergraduate psychology programs, see Box 1).

## Method

We used a modified Delphi technique to systematically elicit anonymous, asynchronous, and iterative input from a range of stakeholders (Humphrey-Murto et al., 2019). We used a consensus process over other methods^3^ of informing updates to the accreditation standards because we wanted to engage the community—which can facilitate the acceptance and uptake of such standards (e.g., Bini & Mahajan, 2016)—and to better understand what the community believes are best teaching practices, because these are not yet established or widely accepted. We used the Delphi method in particular because it can be open to the entire UK psychology community and allows for a large panel of participants from diverse stakeholder groups to provide anonymous responses.

This Delphi study was codeveloped with input from the BPS to ensure that the results could be integrated into their *Standards for the Accreditation of Undergraduate, Conversion and Integrated Masters Programmes in Psychology* (BPS, 2019). We involved the BPS in the planning and execution of this study to benefit from their detailed knowledge of the accreditation standards and because they have the authority to decide how to implement our findings. By engaging them, we hoped to make our study design highly relevant and increase the use of our findings to inform tangible changes (e.g., in line with utilization-focused evaluation; Patton, 2003). The results from this study are not prescriptive to the BPS. The BPS committees responsible for the accreditation standards will follow their internal processes and evaluate the findings from this study along with other forms of evidence. For a thorough explanation of how the BPS was involved, see Supplementary Material A in the Supplemental Material available online.

**Fig. 1:**
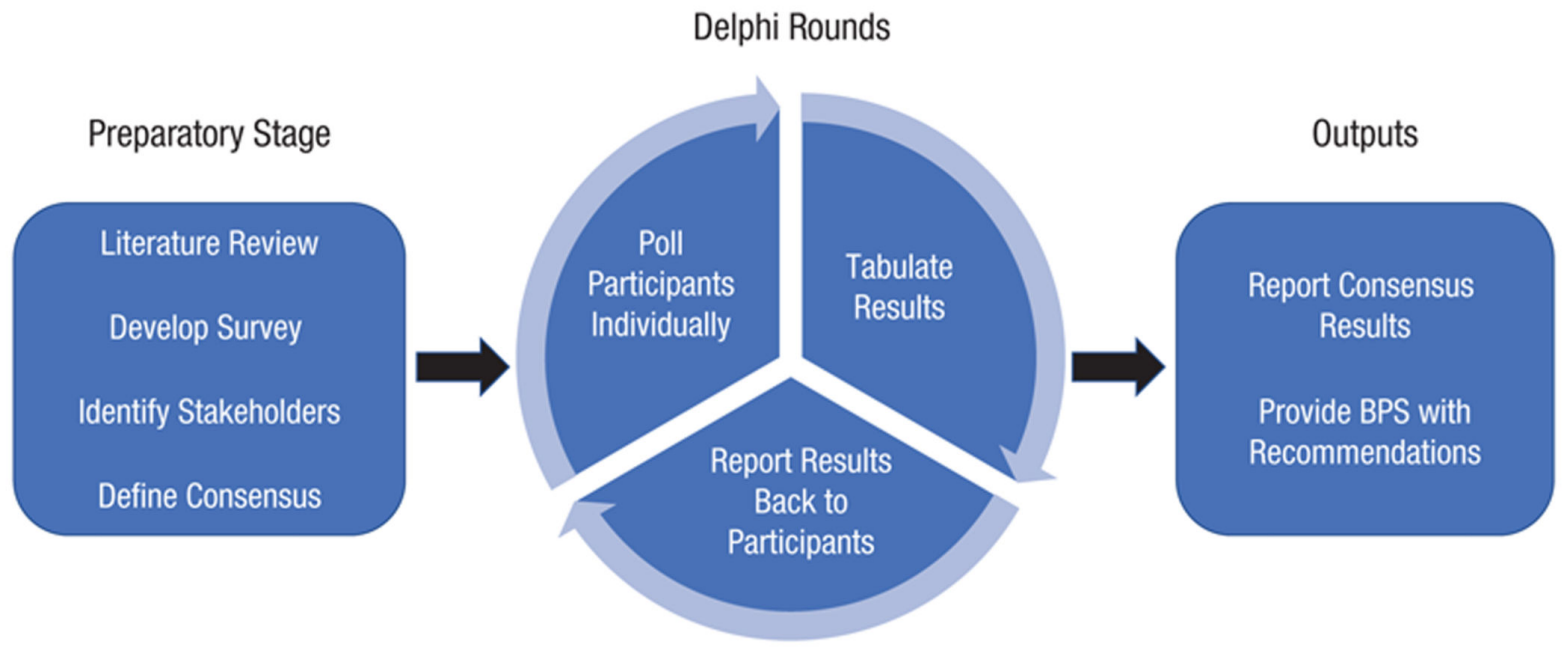
An outline of our modified-Delphi study. A steering committee completed the preparatory stage, participants completed two Delphi rounds, and results were presented to the British Psychological Society Undergraduate Education Committee.

The study included a preparatory stage and two Delphi rounds in which participants rated items and provided open-ended feedback (see Fig. 1). In a modification to the traditional Delphi method, our Delphi did not include an idea-generation round. The preparatory stage was conducted by the Delphi steering committee (see next section). They defined consensus, developed a set of questions that would inform updates to the BPS guidelines, and identified stakeholder groups. The steering committee conducted a nonsystematic literature review^4^ on research-methods education in psychology programs (see Supplementary Material B). This review served as a basis for formulating the survey questions and was shared with participants at the survey’s outset to support informed responses.

The study was approved by the School of Psychological Science Research Ethics Committee at the University of Bristol (ID: 13394). We preregistered a protocol before advertising the study (https://osf.io/5h7bu). Deviations from the preregistered protocol are outlined in Supplementary Material C. This article is reported in line with the ACCORD Checklist (ACcurate COnsensus Reporting Document; Gattrell et al., 2023), which is provided in Supplementary Material D. We report how we determined our sample size, all data exclusions, all manipulations, and all measures in the study.

### Delphi steering committee

We assembled a steering committee to guide this Delphi study. We aimed to include members who met a range of representation criteria. These included representation from the following groups: BPS Undergraduate Education Committee, BPS Partnership and Accreditation Committee, undergraduate psychology program director, quantitative research-methods instructor/expert, qualitative research-methods instructor/expert, psychology researcher, statistician, education expert, UK country other than England, nonacademic psychologist, and relatively recent graduate from an undergraduate psychology program in the UK. With these criteria in mind, the steering committee comprised five members, including the project lead (R. T. Thibault), a BPS representative and undergraduate program lead (R. J. Green), a quantitative psychology research-methods instructor with recent experience as a psychology undergraduate in the UK (J. E. Bartlett), an open-science and pedagogic expert with recent experience as a psychology undergraduate in the UK (M. Pownall), and a qualitative psychology research-methods expert and research-methods instructor (D. Bailey-Rodriguez). We unfortunately did not succeed in achieving representation from a nonacademic person based in psychology. The steering committee communicated with the BPS accreditation operations manager (Patricia Lyons) and chair of the BPS Undergraduate Education Committee (Simon Goodson) to ensure that the study was designed in a way that the results could effectively inform an update to the accreditation standards. Members of the steering committee did not participate in the Delphi.

### Participants

We advertised the Delphi survey via mailing lists and social media. We specifically reached out to the BPS, UK Reproducibility Network, ReproducibiliTea, and The Framework for Open and Reproducible Research Training (FORRT) to help advertise. For a template of the invitation text, see Supplementary Material E. We did not impose a limit on the number of participants.

Participants needed to meet two criteria. First, they needed to be a member of at least one of the following stakeholder groups: (a) student in psychology (undergraduate student, graduate student, or nonstudent who completed an undergraduate degree less than 3 years ago), (b) research-methods instructor in psychology, (c) academic based in psychology, or (d) nonacademic person working in psychology. Participants were instructed to select “option a” if they were an undergraduate or graduate student, regardless of whether they taught or did research, and to select “option b” if they teach or coordinate psychology research methods in an undergraduate or master’s conversion program, regardless of whether they are also an academic or nonacademic psychologist (for verbatim instructions, see Supplementary Material B). Second, participants needed to be either (a) based in the UK (or have been a member of one of the four stakeholder groups in the UK within the past 3 years) or (b) based outside of the UK but be associated with a BPS-accredited psychology program (e.g., the BPS accredits some psychology programs outside the UK).

Before presenting the Delphi items, the survey presented registration questions. These asked the participants whether they primarily conduct qualitative or quantitative research, are a research-methods expert, are an undergraduate program director, and live in the UK and the sector in which they are employed (see Supplementary Material F). No incentives were offered for participation.

### Definition of consensus

Before launching the survey, the steering committee defined consensus as at least 75% of participants in each nonstudent stakeholder group rating an item as “essential” (i.e., between 7 and 9 on the 9-point scale). A 75% threshold is commonly used in Delphi studies (Diamond et al., 2014), and we felt this was a balanced approach. We excluded students from the definition of consensus because they may not have been exposed to many of the concepts presented in the Delphi. The steering committee further made the a priori decision that if a stakeholder group had fewer than 12 participants, we would not require that group to achieve 75% essential responses; instead, we would require that all nonstudent ratings collapsed together reached 75%. We made this decision to avoid a situation in which a very small number of participants are responsible for consensus not being reached.^5^ We performed a sensitivity analysis that required 75% essential ratings from all four stakeholder groups (including students), and it revealed no difference in the items that reached consensus.

### Survey

To conduct the survey, we used the DelphiManager software provided by the COMET Initiative. In Round 1 of the Delphi, participants were asked to rate their level of agreement to 72 items on a scale from 1 to 9 in which 1 to 3 is *not important*, 4 to 6 is *important but not essential*, and 7 to 9 is *essential* (for a screenshot of Round 1, see Fig. S1 in the Supplemental Material). These items ranged from specific content to teach (e.g., effect sizes, qualitative data collection, the replication crisis) to ways of teaching (e.g., evaluation methods) and encouraged resources (e.g., freely available software); for a complete list of items, see Supplementary Material H.

Round 1 comprised eight blocks that each presented between five and 15 items covering the following domains: (a) statistical analyses, (b) quantitative data skills, (c) quantitative research-methods concepts, (d) qualitative research methods, (e) research design, (f) reproducibility and open science, (g) accessibility of resources, and (h) miscellaneous. The blocks were presented in a random order. The items within each block were always presented in the same order (as required by the DelphiManager software). The research design block included an attention-check item that asked participants to select “3.”

The motivation for this study came from the lead author’s (R. T. Thibault) and senior author’s (M. R. Munafò) reflections about the quantitative abilities of psychology graduates and shortcomings in the reproducibility of quantitative psychology research. Items were selected based on previous studies on research-methods education in psychology (e.g., TARG Meta-Research Group, 2022) and with the aim of addressing the knowledge and skills gap that leads to irreproducible research. We aimed to word the items in such a way that the BPS could easily integrate the Delphi results into an updated version of the BPS accreditation standards. Moreover, we aimed to make the items specific enough that someone could assess whether that standard is being met. For example, instead of asking if students should learn to “critically evaluate research,” we asked whether students should learn how to “define and explain questionable research practices (QRPs)” or “cognitive biases (e.g., confirmation bias).” Qualitative research-methods skills were subsequently included in this Delphi upon a recommendation from the BPS representatives. R. T. Thibault drafted an initial list of items that the steering committee and BPS representatives modified and added to.^6^

After participants rated all items in Round 1, the survey asked them to propose additional items that they felt the survey did not include but that they would deem important. The steering committee added some of these suggested items to Round 2 (many were reworded or combined to better match the Delphi structure). To ensure Round 2 did not take too long to complete, only a few suggested items were added. Suggested items were not added if they did not apply to psychology broadly, overlapped substantially with items in Round 1, or were too vague to be meaningfully integrated into the accreditation standards.

All participants who began Round 1 were invited via email to participate in Round 2. Items that reached consensus in Round 1 were removed from Round 2. No items were modified between Round 1 and Round 2. In Round 2, participants were provided with feedback about other participants’ responses from Round 1 and asked to rerate each item. For each item, they were shown the distribution of ratings from each stakeholder group alongside their own response from Round 1 (for a screenshot of Round 2, see Fig. S2 in the Supplemental Material).

Round 1 was open from February 8, 2023, to March 17, 2023. We extended the length of Round 1 because of delays in sending emails to the BPS mailing lists and because we were receiving fewer responses than expected. The initially low response rate may have been caused in part by the University and College Union strikes in February and March 2023. Round 2 was open from April 6, 2023, to April 28, 2023.

### Open-ended questions

Participants were invited to provide open-ended written feedback at several points during the Delphi study. In chronological order, these were as follows: (a) When rating items in Round 1, participants had the option to provide written feedback on each item. (b) After rating all items in Round 1, participants were asked to suggest additional items for Round 2. (c) After completing Round 1, participants were prompted to provide any thoughts they have about the Delphi study. (d) After rating all items in Round 2, participants were asked to give a reason for all their answers that changed rating categories between Round 1 and Round 2. (e) After completing Round 2, participants were prompted to provide any thoughts they have about the Delphi study.

### Analyses

For each item in the Delphi, our main results present the percentage of all participants who rated an item as essential, the mean rating across all participants, and whether the item reached consensus. Our open data (https://osf.io/hpsq4/) include six summary statistics data sheets: one for each stakeholder group individually, one for all nonstudent stakeholder groups collapsed together, and one for all four stakeholder groups collapsed together.

To identify common patterns, one team member (D. Bailey-Rodriguez) applied an inductive thematic analysis to the open-ended textual responses; the analysis was data-driven (Braun & Clarke, 2006). The analysis was based on a critical-realist ontological stance, which assumes that participants’ perspectives, beliefs, and thoughts can be accessed through interpretation of the data to understand their underlying structures, which are thought to be “real” (Willig, 2013). The thematic analysis included a careful reading and rereading of the textual data followed by thorough line-by-line coding. Emerging themes were then identified at the semantic level and reviewed. The themes were subsequently grouped together and given labels. A thematic map was produced, which was further refined, and finally, the report of the analysis was produced (Braun & Clarke, 2006).

Upon viewing the data, we became aware that the DelphiManager software outputs data in a format that does not allow the registration information—except for stakeholder group—to be linked to the ratings of the survey items. Thus, we cannot analyze the participant ratings in relation to their self-reported expertise or current employment (as per Table 1). This data structure also leaves us with registration information for only two samples: (a) all participants who at least completed the registration, even if they did not rate any items or failed the attention check, and (b) all participants who at least began Round 2.

## Results

### Participants

**Fig. 2:**
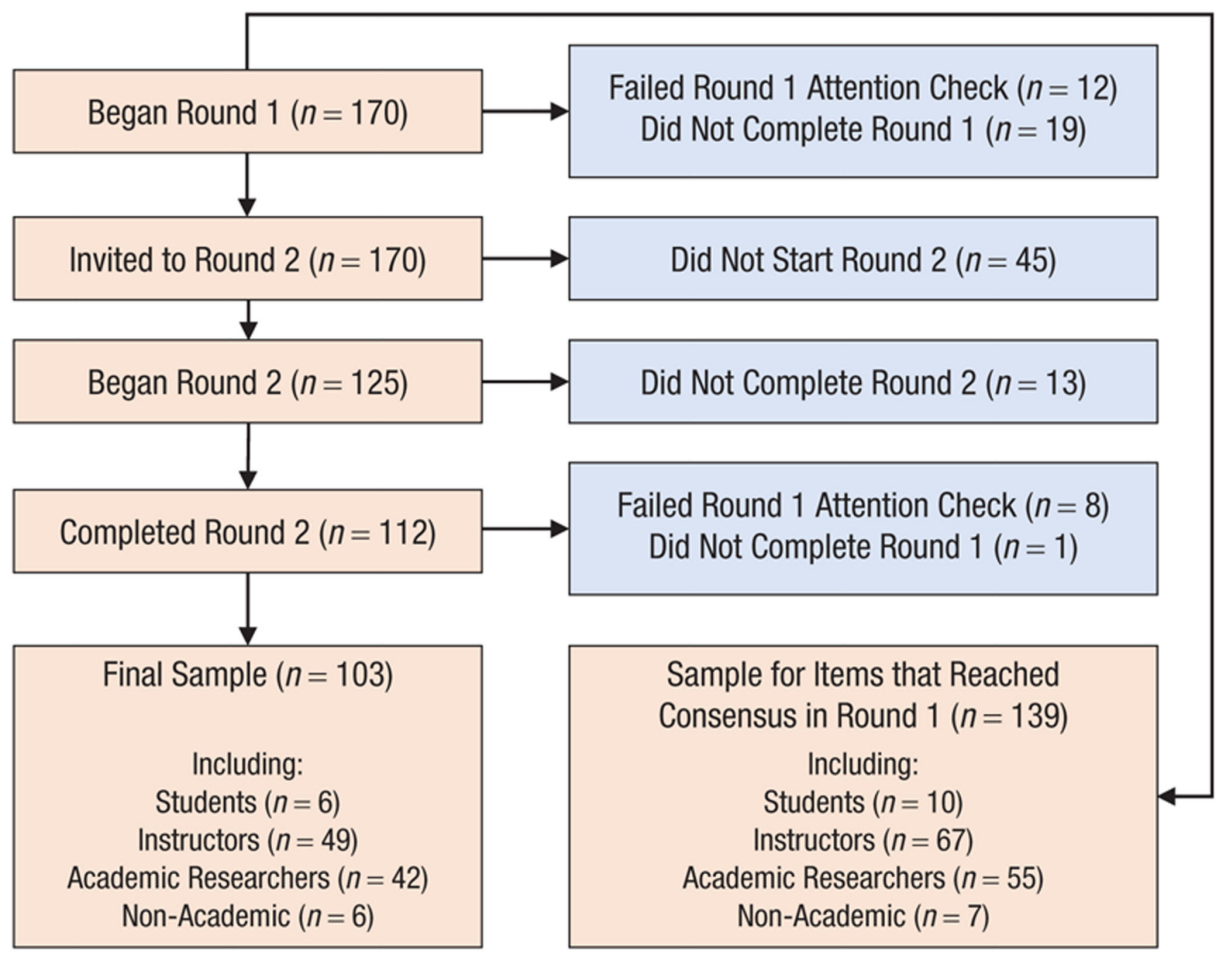
Flowchart of participant inclusion in the final sample. The feedback presented during Round 2 came from the participants in the bottom right box (“Sample for items that reached consensus in Round 1”). Blue boxes indicate participants that were excluded from the final sample.

Figure 2 provides a flowchart of participant inclusion. One hundred seventy participants began Round 1. Items reaching consensus after Round 1 were based on the 139 participants who passed the attention check and completed Round 1. Our final sample comprised 103 participants who passed the attention check and completed both Round 1 and Round 2. The open data include data sheets for each of the final sample, the participants who completed at least one round, and all participants who at least began Round 1.

Table 1 outlines participant characteristics.^7^ The majority of participants who began Round 2 primarily do quantitative research and are employed by universities. They registered for the Delphi using 64 unique email address domain names, which included 55 distinct UK academic domain names (i.e., ending in “.ac.uk”). For all participants who at least registered for the study, these numbers were 86 and 69, respectively.

### Delphi-item ratings

Twenty-six items reached consensus in Round 1 and were not included in Round 2. The steering committee added seven items to Round 2, which thus contained a total of 52 items.^8^

Eight items reached consensus in Round 2. Forty-four items did not reach consensus. Two of the items reaching consensus in Round 2 were added after Round 1 was complete, and two others had already reached consensus among the final sample of 103 participants (but not among the 139 who completed Round 1). Thus, among the final sample, only four items shifted from not reaching consensus in Round 1 to reaching consensus in Round 2. Across all 103 participants, regardless of stakeholder group, the median percentage of participants that rated an item as essential was 74% (interquartile range = 60%–86%). The median of the average (mean) rating across the 78 items was 7.4 (interquartile range = 6.8–7.9).

Table 2 presents the results for each of the 78 items. To explore these results, we recommend opening the spreadsheet available at https://osf.io/57mbd. This spreadsheet includes the verbatim items and domains, which could not easily fit in a pdf but contain important keywords. For example, whereas some items asked if students should learn how to *apply* a technique, others asked if students should learn to *define* a concept.

### Sensitivity Analyses

We performed three sets of sensitivity analyses. We assessed differences in ratings between (a) Round 1 and Round 2, (b) the instructor and academic stakeholder groups, and (c) the initial sample of 170 participants and the final sample of 103 participants. We did not compare results from the students and nonacademic stakeholder groups because they had few enough participants that the comparisons would be uninformative or potentially misrepresentative.

Relatively small differences in ratings occurred between rounds. Of the 47 items included in both Round 1 and Round 2, four went from not reaching consensus to reaching consensus, the median absolute change in the percentage of participants that rated an item as essential was 3.8%, and the median absolute difference in participants’ rating of an item was 0.15. The openended responses revealed several reasons for why participants changed their ratings between rounds, including viewing the ratings of other participants, reflecting further, discussing with colleagues or students, and gaining further knowledge after Round 1. Between instructors and academic stakeholder groups, these differences were six items reaching consensus, 5.3% rating an item as essential, and a 0.24 median rating difference. Only small differences existed between ratings from the sample of participants who at least began Round 1 and the final sample (one item reaching consensus, 1.3% rating as essential, 0.06 median rating difference). For the data in tabular format, see Table S3 in the Supplemental Material.

### Consensus summary results

In this section, we block related items into 13 overarching findings (see Table 3). We conceived these blocks based on whether the items reached consensus and whether they can be interpreted together to help formulate a specific recommendation.^9^ In parentheses, we present the percentage of all participants who rated the items as essential.

#### Consensus largely reached

##### Understanding data

Consensus was reached that students should learn how to identify and categorize different types of data (94%), clean data (88%), anonymize data (86%), and represent quantitative data visually (95%).

##### Research design (general)

Consensus was reached that students should learn how to formulate a research question (99%), design a study to answer a specific research question (97%), explain the difference between a research question and a hypothesis (83%), and identify basic study designs (95%). Consensus was also reached for learning how to create a sampling plan (86%), operationalize all elements of a study (83%), and apply experimental and nonexperimental research designs (82%).

##### Descriptive statistics

Consensus was reached that students should learn how to calculate descriptive statistics (98%), explain the importance of descriptive statistics and how they differ from inferential statistics (95%), and use descriptive statistics effectively before learning to perform inferential statistical tests (87%).

##### Inferential statistics

Consensus was reached that students should learn how to calculate significance tests (96%), regressions (85%), effect sizes (86%), and parameter estimations (e.g., confidence intervals; 79%). Consensus was also reached that students should learn to define and explain probability and randomness (81%) and was almost reached that students should learn to explain the existence of different statistical approaches, including parameter estimation and significance testing (72%).^10^

##### Critical assessment

Consensus was reached that students should learn how to identify and assess ethical issues (99%), assess validity and reliability (91%), define and explain the difference between statistical significance and practical significance (89%), and define and explain the value of exploratory research and how it differs from confirmatory research (85%). Defining and explaining systematic reviews and meta-analysis (70%) did not reach consensus.

#### Consensus reached for some items

##### Qualitative research

Learning to critically appraise qualitative research reached consensus (83%). Several other qualitative items almost reached consensus, including learning to demonstrate understanding of several methods of qualitative data analysis (74%), perform qualitative analysis (73%), use reflexive practice (72%), demonstrate understanding of various qualitative frameworks (71%),^11^ and demonstrate understanding of mixed-methods research (71%). Some qualitative items did not reach consensus, including learning to collect qualitative data (65%), explain the philosophical underpinnings of qualitative research (51%), and apply qualitative frame-works in their own research (50%). Fifteen participants provided open-ended feedback to specific Delphi items on qualitative methods, and 19 participants provided general comments that mentioned qualitative methods. These textual data are analyzed in the article section Theme 1: Important Factors.

##### Reproducibility and open science

Several items related to reproducibility and open science reached consensus, including that students should learn to define and explain sources of bias (91%), cognitive biases (80%), QRPs (85%), generalizability and robustness (89%), research misconduct (90%), and replication studies and reproducibility (82%).

Other items that almost reached consensus include learning to define and explain the replication crisis (75%)^12^ and define and explain data, code, and material sharing (72%). Items more focused on the process of research did not reach consensus, including learning to define and explain the publication process (50%), reward structures in research and academia (44%), pre-registration and Registered Reports (59%), and metaresearch (47%).

#### Consensus largely not reached

##### Advanced analysis techniques

More-advanced analysis techniques did not reach consensus, including learning to perform equivalence testing (34%), perform factor analysis (33%), simulate data (9%), and explain multiverse analyses/many-analyst approaches (17%).

##### Research design (specific)

Items on research designs specific to certain study types did not reach consensus, including learning how to design a survey (65%), apply blinding and randomization (63%), and explain psychometrics (65%). Items about sample size and effect sizes also did not reach consensus, including learning to perform a sample-size calculation for quantitative research (62%), determine a smallest effect size of interest (44%), and explain alternative measures of effect sizes (e.g., probability of superiority; 16%). Learning to select a sample size relevant to the qualitative method being used (76%) almost reached consensus.

##### Approaches to research

Consensus was not reached that students should learn how to consider diverse perspectives when designing a study (69%), define and explain philosophy of science (59%), or define and explain the existence of different statistical frameworks, including frequentist statistics and Bayesian statistics (40%).

##### Computer skills

Consensus was not reached regarding whether students should learn how to use a programming language to manage and analyze data (23%) or use a statistical analysis package with a graphical user interface (66%). Whether research-methods modules should use only freely available software (28%) did not reach consensus. Consensus was almost reached for learning to demonstrate general computer skills for research (73%) and search and collate published research (77%).

##### Module format

No items regarding the format of modules reached consensus, including to never entirely grade with closed-book exams (67%), emphasize skills that transfer beyond an academic research context (67%), have a higher staff-to-student ratio than for non-research-methods modules (70%), provide students with syllabi that include a week-by-week outline of the module contents (70%), or make syllabi publicly available (14%). Actively employing teaching and grading methods known to reduce “statistics anxiety” (75%) almost reached consensus.

##### Final-year projects

Consensus was reached that students should have the option to conduct a qualitative, quantitative, or mixed-methods project in their final-year research (85%). Consensus was not reached on whether students should preregister the quantitative aspects of their final-year project (22%), be allowed to perform a replication as their final-year research project (56%), or be allowed to conduct their final-year project in a team (46%).

### Thematic analysis of the open-ended questions

Participants provided written responses to open-ended questions. These included feedback from 41 participants on 222 specific items, 30 participants for 82 suggested items, and 73 participants regarding 337 ratings that crossed a rating boundary (e.g., from important but not essential to essential). A small number of participants were responsible for large portions of these open-ended responses (see Table S1 in the Supplemental Material). Forty-five participants left a general comment after Round 1, and 21 participants left a general comment after Round 2.

**Fig. 3:**
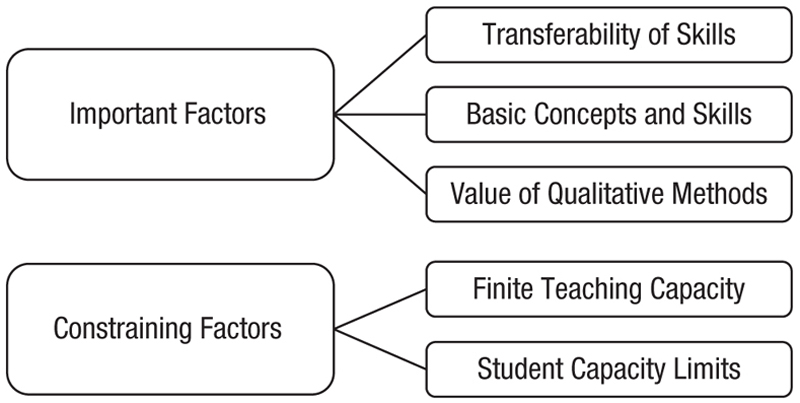
Thematic map with two main themes and their subthemes.

The thematic analysis addressed the following research question: What are important issues to consider when designing the research-methods curriculum? The analysis generated two main themes, “important factors” and “constraining factors,” which encompass the key issues in the design of research-methods curricula. Figure 3 illustrates these themes and corresponding subthemes.

#### Theme 1: important factors

This theme delves into the various factors that were considered to be important to include in the research-methods-curriculum design. The transferability of skills to the workplace, ensuring students understand basic concepts and skills, and the value of qualitative research were prominent issues, and these sub-themes are explored below.

##### Transferability of skills

We identified the transferability of skills to employment contexts as an important factor to be accounted for in the research-methods-curriculum design. For example, a participant said, “A balance is needed to future-proof student skills while focusing on practical understanding of research methods (at UG [undergraduate] level at least).” Another participant wrote, “There should be more consideration given to the … skills that might transfer to other fields, outside academia.”

Most graduates do not pursue an academic career after their psychology degree (Palmer et al., 2021). Thus, teaching research-methods skills that apply beyond academia would be important. This issue was highlighted by a participant:

It would also be great to see research methods taught in a way that emphasizes real-world applications for the students. The vast majority of psychology undergraduates do not go into Psychological research so, while I appreciate the drive to make University level research methods training more consistent with the current state of psychological research, I am concerned that we might be missing the type of data handling skills that students would use in careers that are data focussed but outside of psychological research.

This issue was further exemplified by other participants, for example, “Most psychology undergraduates are not going to be researchers or undertake research in the future” and

It is important for me, as a practicing researcher, to know how to calculate effect sizes, perform a power analysis, navigate publication etc. But is it important for me that students can do these things, given that most of them will not need to in the future?

Participants understood the significance of teaching research methods in ways that are applicable to nonacademic careers.

To this end, participants provided views about the inclusion of transferability skills when teaching research methods that could be applied to nonacademic employment settings, for example, “Teach what is used for research and industry” and “applicability to non-academic settings is important.” Regarding the questions asked in the survey, one participant wrote,

I would have liked to see some questions about what research methods skills are important for students who graduate and don’t get employment in psychology, which is what most of our students do. I feel some of the issue[s] are not relevant to someone who is carrying out research in a non-academic context.

These responses illustrate the importance and value of designing a research-methods curriculum that actively includes research-methods training that can be leveraged in nonacademic employment routes. Nevertheless, the value of teaching research skills that apply both to academic and nonacademic pathways was another important factor for the curriculum. For example, a participant wrote, “Asking students to work as a team is more realistic to how research is conducted in the real world (whether that be academia or in the public or private sector).”

Furthermore, for psychology graduates intending to follow academic careers, our analysis suggests that it is also important to teach transferable skills that reflect what happens in real-world research, for example, “we falsely give students the idea research is a fairly solo/small homogenous (student) group activity whereas the truth in many fields related to psychology is that it needs multidisciplinary teams including methodologists and lay advisors.” For further illustration, in relation to the survey question “the publication process,” another participant wrote “very important when it comes to following an academic path.” In addition, in relation to the survey question “students should be allowed to perform a replication as their final year research project,” another participant stated, “This is more representative of actual psychology research, and in line with open science practices.” The comments demonstrate the importance of transferable skills taught in line with academicemployment routes.

However, there was some tension in which specific skills should be taught as important for transferability. For example, a participant stated “SPSS is still very important in government, business, and charities,” and another wrote, “SPSS is in declining use. Psychology departments do students a disservice by continuing to use software that is not used in other disciplines or in industry.” Despite the disparity in the specific transferable skills some participants suggested, it remains important that skills learned in the research-methods curriculum have applicability to employment contexts in both academic and nonacademic pathways.

##### Basic concepts and skills

We identified that ensuring students understand basic concepts and skills was another factor deemed to be essential in the research-methods-curriculum design. For example, participants stated, “I believe students should learn the basics of all research methods” and “We need to focus on students getting the foundational methods knowledge.”

There were concerns about the increasing number of topics and skills being taught in the research-methods curriculum at this level, to the detriment of students’ understanding and ability to apply these properly. To illustrate, a participant wrote, “There is quite a focus on teaching UG students more and more topics, and using potentially more technical platforms. I would also like to see emphasis on improving students’ understanding and ability to apply foundation concepts,” and another stated, “At the UG level what we need to be doing is covering LESS but with greater rigour and confirmation that students can actually USE and UNDERSTAND what we have taught them.” The responses demonstrate the importance of making sure that the research-methods curriculum includes space and time for the teaching of concepts and skills considered to be foundational and to avoid the overload of an ever-increasing number of topics and skills when basic understanding of concepts and skills seems to be lacking.

The negative impact of the increased taught concepts and skills is illustrated in the following participants’ statements: “I end up essentially doing the major parts of their [final year] project for them” and “We teach research methods in 1st year and 2nd year and they arrive at their 3rd year dissertations knowing practically nothing, despite often doing well on earlier exams.” Not ensuring that students comprehend basic research-methods concepts and skills in the earlier years appears to have a negative knock-on effect on their ability to understand and conduct their final-year projects.

Furthermore, the importance of ensuring a basic understanding of concepts and skills also applies when teaching students to use analysis software. For example, participants wrote, “For all of these [software skills] it’s necessary to explain why we do these tests, what they mean, give real world examples” and “For too long have psychology departments ‘taught SPSS’ instead of teaching statistical analysis.” It is important that students have foundational understandings so that when the time comes to use analysis software, they comprehend what they are doing and why. This issue is further exemplified by the following quote: “Should we address the problematic tendency to emphasise which stats package dialogue boxes to tick at the expense of teaching a deep understanding of what the analysis actually does?” This comment illustrates the importance of designing the research-methods curriculum in a way that ensures students have a solid understanding of basic research concepts and skills.

In addition, our analysis found that ensuring students develop the ability to think critically was another important foundational concept for developing their understanding and appraisal skills. For example, in this context, a participant stated, “I would like the field to be careful about ensuring that students are not simply directed towards a checklist approach,” and another wrote, “We ought to be teaching ways to understand, critique, appraise and undertake research.”

The value of ensuring students learn to evaluate research transcends the particular topic under critical review, as exemplified by some participants: “Not everyone will want to use qualitative research methods; but everyone should be able to evaluate published qualitative research”; “I think they need to be made aware of the limitations of quantitative research, which they tend to think of as sacrosanct”; “It’s essential for students to understand that this [the publication process] is not a neutral process and factors in the process create a biased literature”; and “I’d prefer them to know why replication is relevant and why it’s become a fixation as opposed to other issues.” These responses demonstrate the importance of ensuring students develop critical-thinking skills as foundational in all research-related contexts and integrated into the curriculum as such.

On the whole, the findings illustrate the importance of designing a research-methods curriculum that ensures students understand basic research concepts and skills, including in teaching analysis software and in the development of critical-thinking skills.

##### Value of qualitative methods

Our analysis identified the value of qualitative research and its inclusion in the research-methods curriculum as another important factor. Illustrating this finding, participants wrote, “Students need more qualitative methods input, this is crucial to ensuring the quality of qualitative research and of future training”; “Qualitative analysis is profoundly important”; and “I have rated most of these [survey items relating to qualitative research] as essential.”

In addition, it was recognized that the survey further problematized the perception that qualitative methods are not as valuable as quantitative methods, for example, “It is interesting that even in this study, a greater emphasis is placed on quantitative over qualitative skills (judged by the number of questions). I believe these should be given equal weight.”

Furthermore, our analysis suggests that qualitative research is currently viewed as having less importance than quantitative methods, but several participants wrote that it should have the same value status in the research-methods curriculum. For example, participants stated, “QUANTitative and QUALitative research methods should be given equal attention by staff and students”; “There is an imbalance here on the quant to qual methods - both are equally important and should be given equal importance in undergraduate study”; and “Qualitative research methodology should be given the same emphasis as quant so that students appreciate the relative value and appropriate applications.” These comments indicate that participants felt that qualitative methods should hold equal value to quantitative research in the curriculum and that this value should be held by research-methods instructors and students.

Another important factor that stems from recognizing the value of qualitative research is that some qualitative concepts are directly applicable to quantitative methods. This was particularly salient when participants were responding to the survey question, “Explain the philosophical underpinnings of qualitative research,” for which some stated, “And of quantitative research!”; “Students need to know about the theoretical and epistemological underpinnings of research full stop, not just qual”; and “Should do this in all research.”

Overall, qualitative methods are considered to be valuable and an important factor to include in the research-methods-curriculum design, alongside the view that they should hold equal status to quantitative research methods.

#### Theme 2: constraining factors

This theme unpacks several factors that were considered to be constraining in the design of research-methods curricula. Finite teaching capacity and student-capacity limits were significant issues. These subthemes are examined below.

##### Finite teaching capacity

Our analysis identified that the finite amount of time to teach the many research concepts and skills was considered to be a constraining factor. For example, in response to the survey items, participants stated, “The study doesn’t really take into consideration what is achievable in a given timeframe and whilst a lot of ideas and concepts are really important I am not sure how you would fit them all in”; “A lot of my observations of things being ‘important but not essential’ are really shaped by my understanding of the time constraints in our teaching”; and

.’s a bit too easy to say “everything is really important!” (certainly the RM sample looked like almost everyone was saying “9” for loads of the items), when actually there’s a finite amount we can teach our students and expect them to learn in Y1 and Y2 of an UG degree.

Despite participants wanting to support the value of many of the survey items proposed, it seems responses also took into account the finite amount of capacity available to teach these concepts even though this was not specifically asked as part of the survey. This finding reflects the importance of taking this constraining factor into account when designing the research-methods curriculum.

Concerns regarding finite teaching capacity also extended to the teaching of particular research skills, such as mixed methods, for example, “Mixed methods is often not feasible within the timeframe allowed; it may also require careful staff allocation which could create lack of equality of supervision,” and to learning to use a programming language, for example, “This entails considerable staff time.” These responses illustrate the need for careful consideration as to what concepts and skills should be included in the research-methods curriculum given the finite amount of teaching capacity.

The impact of what can be taught in the given amount of time available for research methods can be a negative one, both on students and on instructors, as exemplified by the following participants. One wrote, “In my experience, most students have absolutely no idea of what they’re doing and have no capacity or time to be trained, either on their part or mine because I am so wildly overwhelmed,” and in response to the survey item, “Have a higher staff to student ratio than for non-research methods modules,” another participant wrote, “Definitely would be good, but hard to implement and also ties closely together with how many taught hours are involved (labs/lectures) and what that means for teaching loads.”

On the whole, the finite capacity for teaching research methods is a constraining factor on the concepts and skills that could be included in a research-methods curriculum, especially in view of the negative impact that overfilling the curriculum can have on students and instructors.

##### Student-capacity limits

Our analysis also found that restrictions in student capacity was a constraining factor regarding what is feasible to include in a researchmethods curriculum. This finding is illustrated by a participant’s comment: “I think at the moment we’re trying to turn all our UG students into PhD level R users and researchers (too much!).”

Participants seemed to feel that there are limits to what students can learn within the degree time frame, for example, “As much as I use and like R, I’m not sure it’s reasonable to make all students learn to code” and “I don’t think it’s necessary for students to apply multiple types of research designs; there is only so much time in a degree. I think it’s more important to know about it.”

Furthermore, participants thought that certain research skills were too advanced for inclusion in the research-methods curriculum. Mixed methods were one such concept, for example, “I worry that mixed methods can be too complex/large scale to be achievable,” “Mixed methods reports are too complicated for final year research,” and “Mixed methods is less important than good qual and good quant and throws up some real issues for final year projects.”

Participants also considered learning to use a programming language to be too advanced for students: “Many students may be overwhelmed by learning a programming language (which may increase students leaving courses),” “This would put off and disadvantage many students,” and “Sadly, we struggle to get our students to get to grips with SPSS; when have tried to teach R, it has been even more difficult.” These responses indicate that designing a research-methods curriculum requires careful consideration regarding the concepts and skills that fit the capacity of students at this level.

Nevertheless, the option for students to specialize in more-advanced research concepts and skills was considered to be a potential way to overcome this constraining factor: “I’d like to see research methods training that starts more broadly and becomes more specific; offering students a choice of where to specialize.” In response to the survey item, “Use a programming language to manage and analyse data,” some participants stated, “We can certainly teach why this would help and encourage those who’re interested/able to explore it. … This should be an optional skill” and “This is important but not essential for all students. They should be given an opportunity to learn this as part of an elective module.”

Overall, student-capacity limits are also a constraining factor that should be considered when designing research-methods curricula. There are limits to what students can learn within the degree time frame, and some research concepts and skills were thought to be too advanced. Providing the option for students to learn more-advanced concepts and skills could be a possible solution.

## Discussion

### Summary of results

Consensus was reached for 34 items. These items spanned topics, including data skills, general research design, descriptive statistics, inferential statistics, critical assessment of research, and to some extent, qualitative methods, reproducibility, and open science. Consensus was not reached for 44 items. These items spanned advanced analysis techniques, specific research designs, approaches to research, computer skills, module formats, and final-year projects. A qualitative analysis of openended responses highlighted the importance of understanding basic concepts, valuing qualitative research methods, and learning transferable skills while also acknowledging limits on how much material can fit in an undergraduate program and how much students can absorb in this finite time period. Taken together, these results can provide valuable information for instructors, program directors, and organizations that develop accreditation standards.

### Relation to the literature

The consensus results partially overlap with the content that appears on publicly available curricula for quantitative research methods in psychology programs (TARG Meta-Research Group, 2022). For example, items such as descriptive statistics, inferential statistics, and critical evaluation were prevalent across curricula and also rated highly in this Delphi study. On the contrary, items such as effect sizes, confidence intervals, data cleaning, practical significance, and replication were less prevalent in the curricula^13^ but were highly rated in this Delphi study. Adding these topics to accreditation standards presents one mechanism to encourage their adoption. Almost all the curricula mention SPSS; however, learning to use a statistical analysis package with a graphical user interface did not reach consensus in our study. Our qualitative analysis further suggests that participants were concerned that students learn how to “point-and-click” in SPSS rather than gain an understanding of the analyses for which they are using the software. Comparing curricula with accreditation standards—and with the results of this Delphi study—can help understand whether the educational content of psychology programs aligns with community expectations.

Several qualitative items had lower ratings than quantitative items, reflecting a previously observed trend. For example, qualitative methods appear to be underrepresented in curricula and perceived as an alternative and “lesser” approach to quantitative methods in UK psychology programs (Gibson & Sullivan, 2018; Hugh-Jones et al., 2012). Interviews with psychology instructors also suggest that some programs would need additional instructor expertise to effectively teach and supervise qualitative research methods (Wiggins et al., 2016). Indeed, almost all participants in our study (96%) agreed that research-methods instructors should be given time and support to improve skills they plan to teach, including qualitative methods. Given the importance the BPS places on qualitative methods (e.g., the Qualitative Methods in Psychology Section of the BPS), the prominence of qualitative methods in the accreditation standards could be raised.

Transferability of research-methods skills emerged as a theme and appears relevant given the diverse career paths that psychology graduates follow. A recent report in which data from the Higher Education Statistics Authority were analyzed found that “there is no common career path for psychology graduates, as they go on to work in a broad array of roles and settings” (Palmer et al., 2021). The report further stated that only about 6% of graduates become registered professionals in psychology and that many go into roles in the health sector, retail, administration, public relations, marketing, and human resources. In these roles, a solid foundation in qualitative and quantitative skills likely trumps the ability to perform inferential statistical tests. Even in careers in which inferential statistics are necessary, such as academic research, foundational data skills are also necessary. For example, a recent article calling for UK psychology education to emphasize data skills demonstrated that to analyze a realistic quantitative data set in psychology, data wrangling (e.g., cleaning and structuring data) accounts for about 80% of the steps, and statistical procedure accounts for only 20% of the steps (McAleer et al., 2022).

### Recommendations

As per our study objective, we provided the BPS Undergraduate Education Committee with nine core recommendations for updating their accreditation standards (see Supplementary Material I; also summarized in Box 2). We developed these recommendations by considering the combination of the consensus summary results and the thematic analysis. Although we would recommend that all 34 items that reached consensus be considered for inclusion in a research-methods curriculum, we binned items into nine recommendations that integrate the qualitative data and hopefully facilitate the implementation of the recommendations.

Ratings were very high, and consensus was reached for data skills, basic research design, descriptive statistics, and inferential statistics. Almost 90% of participants rated it essential that students learn to use descriptive statistics effectively before learning to perform inferential statistics. Our qualitative analysis also highlighted the need for students to master foundational quantitative and qualitative skills rather than attempt to perform analyses that they understand poorly. These findings challenge the NHST-centric approach taken in many research-methods curricula and suggest that the psychology community should place importance on ensuring students develop a deeper understanding of the research skills they are using and why they are using them.

Open-ended comments raised the point that students should learn how to answer a research question and focus on fewer technical abilities. With this in mind, researchmethods education could adopt a problem-solving approach by teaching students how to ask a clear question, design an effective research plan, identify what data are needed to answer their question, and how that data could be collected (e.g., the problem-plan-data-analysisconclusion model, as suggested by Spiegelhalter, 2019).

Critical assessment is a pillar throughout the 2019 BPS accreditation standards and mentioned in almost all curricula assessed in a previous study (TARG Meta-Research Group, 2022), but specific concepts and tools are generally not outlined. Updated accreditation standards could include specific items, such as learning about replication and sources of bias, to create a more structured approach for critically assessing the psychology literature and other forms of information. This topic is linked to integrating the principles of open science into undergraduate education, which others have encouraged (e.g., Pennington, 2023; Pownall et al., 2023).

Forty-four items did not reach consensus. These items spanned topics including module format, final-year projects, computer skills, approaches to research, and advanced analysis techniques. Many of these items received a high level of agreement but fell short of consensus. There was not consensus against teaching these items.

### Limitations

Our study design entails limitations on the claims we can make and how they can be interpreted. First, Delphi studies assess the opinions of a community. They do not establish what educational content is most effective. For example, some participants may simply provide low ratings for items they are unfamiliar with. In our results, for example, consensus was reached for learning about practical significance. However, related concepts that participants may be less familiar with received low ratings (e.g., alternative measures of effect sizes and smallest effect sizes of interest). We hope to have mitigated this limitation by providing the option “unable to rate,” which was used for 3% of ratings.

Second, the format and content of our Delphi was specifically designed in relation to the BPS accreditation standards. This meant that we selected items and phrased them in such a way that our results could be easily integrated into these standards. In this sense, we did not present items that challenged core components of the standards (e.g., the inclusion of a final-year project^14^) or content that already exists in the standards that we believed participants would be unlikely to disagree with (e.g., “critical evaluation”). Many Delphi studies include an initial idea-generation round in which participants are asked to suggest items before they see or rate any item. Some Delphi studies also include an item-prioritization round, in which participants rank the items that reached consensus. Because of limited resources and time constraints, we did not include these rounds.

Third, we targeted four stakeholder groups but achieved a substantial number of participants in only two of these groups. These two groups overlapped substantially, given that most research-methods instructors are likely also academic psychologists, and their ratings were relatively similar. Very few students and nonacademic psychologists participated even though the BPS sent invitations to relevant mailing lists (e.g., Student Ambassadors, Psychological Professions Network). Thus, in Round 2, most participants saw the ratings from only a few students and nonacademic psychologists (which may have comprised an unrepresentative sample) and from one other group with similar responses to their own group. This combination of factors may have contributed to the limited changes to ratings between Round 1 and Round 2^15^ and also resulted in a failure to capitalize on this strength of the Delphi technique. The steering committee also lacked a nonacademic psychologist and a very recent graduate, which may have resulted in the content and wording of Delphi items—and thus the results—being less applicable to nonacademic contexts and student perspectives. The lack of engagement from nonacademic psychologists and students may arise because of a combination of a lack of interest from these stakeholder groups and a further distance from the steering committee who ran this study.

Fourth, our sample was likely biased toward quantitative psychologists and people who are highly interested in or opinionated about research methods. A majority of the participants reported primarily using quantitative methods, and few reported primarily using qualitative methods. This distribution of participants—which may or may not reflect the distribution of the psychology community in the UK—could be responsible for the generally higher ratings for quantitative items compared with qualitative items. By design, our study also reflects only the views and priorities of the UK psychology community.

Fifth, the study was originally conceived to ask only about quantitative issues and was thus weighted toward quantitative methods. Qualitative methods were included upon the suggestion of BPS representatives. We did not include items asking about the proportion or ordering of teaching quantitative versus qualitative methods. Several participants provided feedback expressing concern regarding this quantitative-qualitative imbalance and stated that some qualitative items were poorly worded. This imbalance may have affected the distribution of ratings between the quantitative and qualitative items.

Sixth, participants may have overlooked conditional words that preceded some items. For example, one Delphi item asked if students should learn how to “*calculate/ perform* significance tests,” and another asked if students should learn to “*define and explain* systematic reviews and meta-analysis.” This oversight could have lowered ratings for some items because participants may have thought that they were being asked if students needed to learn how to “perform” a meta-analysis, for example.

We were aware of the limitations of the modified-Delphi format we used before beginning the study and deemed them acceptable. The shortcomings of sampling bias and limited student and nonacademic engagement limit the generalizability of our results to the psychology community at large. Nonetheless, our data and findings provide a resource that can help inform accreditation bodies, program directors, and module instructors about what the UK academic community believes is essential for undergraduate psychology students to learn.

Our study also had several strengths. The Delphi method is a recommended approach to produce guidelines on topics for which data are scarce and expert opinion or community opinion is the best available evidence. By opening participation in the Delphi to the UK psychology community at large, identifying key stakeholder groups, and sending targeted invitation emails to those groups, we avoided sampling bias that would arise through other methods, such as selecting a panel of experts to complete the Delphi. Our mixed-methods approach also provides a robust understanding of participants’ opinions. We also received responses from more than 100 members of the UK psychology community from more than 50 UK universities, including 20 program directors. We also worked with representatives from the BPS to ensure that we selected items and worded them in a way that facilitates integration into updated accreditation standards. Finally, all the raw data, summary data sheets, and analysis code are publicly available for others to explore.

## Conclusion

Our study provides data sets, both quantitative and qualitative, on the research-methods skills that UK-based instructors and academic psychologists deem essential for undergraduate psychology students to learn. Our findings suggest widespread agreement that researchmethods education in undergraduate psychology should emphasize foundational skills in research design, data handling, statistics, qualitative methods, and critical assessment while providing students with transferable skills and not overloading them with advanced techniques. Organizations that create educational standards for psychology programs—such as the BPS—can draw on our findings to help develop broadly accepted and clear-cut expectations for research-methods education. Such initiatives could foster cohorts of graduates with an established set of competencies tuned for the contemporary world.

## Supplementary Material

Supplementary material

## Figures and Tables

**Table 1:** Characteristics of Participants Who at Least Began Round 2

Participant characteristic	*N* = 125
Expertise	
Primarily do quantitative psychology research	77
Primarily do qualitative psychology research	25
Do similar level of quantitative and qualitative research	20
Self-identified as research-methods expert	40
Undergraduate program director	20
Primary employer	
University (equal teaching and research)	57
University (primarily teaching)	36
University (primarily research)	13
Graduate student	7
Clinical practice	4
Other	4
Undergraduate student	3
Industry	1
Relation to UK psychology	
Live in UK	123
Associated with BPS-accredited program outside the UK	1
Not associated with the UK	1

Note: Participants could select multiple responses for the “expertise” characteristic. BPS = British Psychological Society.

**Table 2:** Rating and Consensus for the 78 Delphi Items

Item	Domain	Rated essential (%)	Mean rating	Consensus
Formulate a research question	Design	99	8.8	1
Identify and assess ethical issues	Design	99	8.6	1
Descriptive statistics	Stats	98	8.7	1
Design a study	Design	97	8.5	1
Time and support to improve instructor skills	Misc	96	8.5	1
Significance tests	Stats	96	8.4	1
Represent data visually	Data	95	8.4	1
How descriptive statistics differ from inferential statistics	Quant	95	8.3	1
Identify and categorize different types of data	Data	94	8.4	1
Sources of bias	OS	91	8.0	1
Research misconduct	OS	90	8.0	1
Generalizability and robustness	OS	89	7.8	1
Practical significance	Quant	89	8.1	1
Clean data	Data	88	8.0	1
Use descriptive statistics before inferential statistics	Data	87	8.0	1
Create a sampling plan and data collection plan	Design	86	7.9	1
Effect sizes	Stats	86	8.0	1
Methods to assess statistical assumptions	Stats	85	7.9	1
Questionable research practices	OS	85	7.7	1
Regression	Stats	85	8.0	1
Critically appraise qualitative research	Qual	83	7.7	1
Explain research question versus hypothesis	Design	83	8.2	1
Follow accepted reporting guidelines	Design	82	7.8	1
Replication studies and reproducibility	OS	82	7.7	1
Probability and randomness	Quant	81	7.7	1
Parameter estimation (95% confidence intervals)	Stats	79	7.7	1
Identify basic study designs	Design	95	8.2	2
Assess validity and reliability	Design	91	7.8	2
Anonymize data	Misc	86	7.9	2
Option for qual, quant, or mixed-methods final-year project	Misc	85	8.0	2
How exploratory research and confirmatory research differ	Quant	85	7.8	2
Operationalize all elements of a study	Design	83	7.7	2
Apply experimental and non-experimental research designs	Design	82	7.7	2
Cognitive biases	OS	80	7.7	2
Search and collate published research	Misc	77	7.4	No
Select a sample size for qualitative research	Qual	76	7.4	No
Employ methods known to reduce “statistics anxiety”	Misc	75	7.6	No
The “replication crisis”	OS	75	7.5	No
Demonstrate understanding of qual data analysis methods	Qual	74	7.3	No
Perform qualitative analysis	Qual	73	7.3	No
Demonstrate general computer skills for research	Data	73	7.3	No
Data, code, and material sharing	OS	72	7.1	No
The existence of different statistical approaches	Quant	72	7.2	No
Use reflexive practice	Qual	72	7.5	No
Demonstrate understanding of mixed-methods research	Qual	71	7.1	No
Demonstrate understanding of qualitative frameworks	Qual	71	7.2	No
Higher staff-to-student ratio for research-methods modules	Resources	70	7.2	No
Provide syllabi with week-by-week module outline	Resources	70	7.2	No
Systematic reviews and meta-analysis	OS	70	7.0	No
Consider diverse perspectives when designing a study	Design	69	7.1	No
Emphasize skills that transfer beyond academic research	Resources	67	7.1	No
Never entirely graded using closed-book exams	Resources	67	7.1	No
Use graphic user interface statistical analysis package	Data	66	6.7	No
Collect qualitative data	Qual	65	7.2	No
Design a survey	Design	65	7.0	No
Psychometrics	Quant	65	6.8	No
Apply blinding and randomization	Design	63	6.9	No
Perform sample size calculations	Design	62	7.0	No
Philosophy of science	OS	59	7.0	No
Preregistration and Registered Reports	OS	59	6.8	No
Allowed to perform a replication as their final-year project	Misc	56	6.6	No
Explain philosophical underpinnings of qual research	Qual	51	6.7	No
Apply qualitative frameworks	Qual	50	6.4	No
The publication process	OS	50	6.3	No
Metaresearch/metascience	OS	47	6.4	No
Allowed to conduct their final-year project in a team	Misc	46	5.8	No
Determine a smallest effect size of interest	Design	44	6.5	No
Reward structures in research and academia	OS	44	6.5	No
The existence of different statistical frameworks	Quant	40	5.9	No
Equivalence testing	Stats	34	6.0	No
Factor analysis	Stats	33	5.8	No
Use only freely available software	Resources	28	5.4	No
Use a programming language	Data	23	5.3	No
Preregister quantitative aspects of final-year project	Misc	22	5.1	No
Multiverse analyses/many-analyst approaches	OS	17	4.8	No
Alternative measures of effect sizes	Quant	16	4.9	No
Make syllabi publicly available	Resources	14	4.5	No
Simulate data	Data	9	4.4	No

Note: Items are ordered by the column Consensus and then Rated Essential (%). Many items have been paraphrased so they can fit in this table. The domains have been shortened (stats = statistical analyses; data = quantitative data skills; quant = quantitative research methods concepts; qual = qualitative research methods; design = research design; OS = reproducibility and open science; resources = accessibility of resources; misc = miscellaneous). Full verbatim descriptions of the items and domains are available in the following spreadsheet: https://osf.io/57mbd. The rating scale ranges from 1 to 9, in which 1 to 3 is *not important*, 4 to 6 is *important but not essential*, and 7 to 9 is *essential*. The Consensus column contains a value of 1 if consensus was reached in Round 1, 2 if reached in Round 2, and “no” if consensus was not reached. Items that reached consensus in Round 1 have the columns Rated Essential (%) and Mean Rating taken from Round 1 (because these questions were not included in Round 2).

**Table 3:** Recommendation Topics Derived From Consensus Results

Consensus largely reached	Consensus reached for some items	Consensus largely not reached
Understanding data	Qualitative methods	Advanced analysis techniques
Research design (general)	Reproducibility and open science	Research design (specific)
Descriptive statistics		Approaches to research
Inferential statistics		Computer skills
Critical assessment		Module format
		Final-year projects

## Box 1 Contextual Information Regarding UK Undergraduate Psychology Programs and British Psychological Society Accreditation

There are several notable differences between undergraduate psychology programs in the UK and in other countries, such as the United States.

### Terminology

In this article, we use the term “program” to denote the entirety of the education needed to earn an undergraduate degree in a particular field of study (often called a “course” in the UK). A program consists of multiple “modules” (which are often called “courses” or “classes” in the United States). A module typically lasts 1 term and focuses on a specific subject.^1^

### Program structure

UK undergraduate programs are generally 3 years in length (except for in Scotland, where they are 4 years). Students are expected to learn quantitative and qualitative skills. In the final year of the program, students must conduct a “substantial piece of research … [that] typically involves the collection of original empirical data from participants” (BPS, 2019). Compared with students in US psychology programs, students in UK programs generally have fewer opportunities to take elective modules outside of psychology or select from optional modules within psychology.

### BPS accreditation

The BPS accredits the vast majority of UK undergraduate psychology programs (771 programs across more than 200 Higher Education Institutes). For UK-based applicants to enroll in postgraduate training as a professional psychologist (e.g., as a clinical psychologist), they need to have completed a BPS-accredited undergraduate program (or BPS-accredited postgraduate conversion program).^2^ This accreditation landscape creates a circumstance in which the BPS can influence the content and structure of the psychology programs that approximately 24,000 students are admitted into each year (Palmer et al., 2021).

### Accreditation content

The BPS accreditation standards expand on the UK Quality Assurance Agency (QAA) for Higher Education (2023) benchmark statement, which defines what can be expected of a graduate and exists for several disciplines, including psychology. Both the QAA benchmark statement and BPS accreditation standards are updated every few years (the previous versions for both were published in 2019). The BPS standards consist of a 32-page document that outlines eight overarching standards encompassing program design, content, admissions, governance, and resources, among others. The program content requirement includes seven core curriculum areas: biological psychology, cognitive psychology, developmental psychology, individual differences, social psychology, conceptual and historical issues in psychology, and research methods. To earn BPS accreditation, a program must meet these standards, including coverage of the core areas. In this article, we focus only on research methods. The BPS (2019) states that they take a partnership rather than policing approach to accreditation.

Although the present study was designed specifically to inform the BPS accreditation standards, we feel that the findings and recommendations provide an evidence base that psychology instructors, program directors, and accrediting bodies in other countries can apply.

## Box 2 Recommendations for Research-Methods Education in Undergraduate Psychology Programs

1. Require a strong understanding of **data and quantitative data skills**.
2. Emphasize general skills in **research design**.
3. Prioritize a solid foundation in **descriptive statistics**.
4. Provide students with a **framework for critical assessment** of research claims.
5. Raise the prominence of **qualitative methods** throughout the accreditation standards.
6. For **inferential statistics**, require that **parameter-estimation techniques**, such as confidence intervals and effect sizes, are taught alongside significance testing.
7. Give precedence to teaching **foundational research-methods skills** (as outlined in Recommendations 1–5).
8. Promote content that elucidates how research-methods skills **transfer beyond academia**.
9. **Enable Recommendations 1–8** by encouraging research-methods education throughout the program, focusing on fewer skills in greater depth, and offering optional modules for more advanced research-methods skills.

## Footnotes

1 This paragraph was copied from TARG Meta-Research Group (2022) and edited to match journal style.

2 This is known as the Graduate Basis for Chartered Membership (GBC).

3 Other nonconsensus approaches are also possible. We could have collected and curated recommendations from experts. However, bias emerges regarding who is deemed an expert, and the lack of community input could reduce uptake. We could have also systematically reviewed teaching practices or run an experiment to identify good teaching methods. If we took one of these approaches, we would still need community input to define the learning outcomes that “best practice” leads to. These methods also look only at past data. They may be less informative about what educational material and methods of teaching the community believes will be important going forward.

4 R. T. Thibault drafted a literature review based on the TARG Meta-Research Group (2022) article he coauthored. The draft was then shared with all members of the steering committee, who suggested several additional articles to include. We also used a snowball method in which we scanned for relevant citations in the publication we reviewed. We did not conduct a systematic review or use a formalized search method.

5 For example, to prevent a situation in which three of 10 participants in one stakeholder group rate an item as nonessential (which would preclude consensus) but the vast majority of 100 participants across all other stakeholder groups rate an item as essential.

6 R. T. Thibault also sought input from his research group: the Meta-Research Innovation Center at Stanford University. Round 1 was piloted by steering committee members R. T. Thibault and J. E. Bartlett and a colleague, Charlotte Pennington, a lecturer in psychology. We had only a short time frame to prepare and run the study, and these people were able to pilot promptly. Only R. T. Thibault piloted Round 2.

7 We preregistered that we would remove participants who responded that they were not associated with the UK. Four participants from our initial sample and one from our final sample selected this response. However, because the participant registration information could not be linked with the participant ratings (as explained in the Analyses section), we could not exclude these participants’ ratings.

8 72 (Round 1 items) – 26 (items that reached consensus) – 1 (attention check) + 7 (new items) = 52 (Round 2 items).

9 Some of these blocks overlap with how the survey was presented: in eight blocks, or “domains.” However, whereas those domains were based only on the overarching topic of the items, the blocks in this section incorporate both the topic and the consensus results.

10 In the Consensus Summary Results section, we use the term “almost reached consensus” for all items with more than 70% essential ratings but did not meet our preregistered definition of consensus. This decision was not preregistered. We feel it helps avoid a strict dichotomization.

11 Including the term “various” in this item may have prevented some participants from rating it as essential.

12 Some items had more than 75% essential ratings when collapsed across all participants but did not have more than 75% agreement within each stakeholder group and thus, did not meet our definition of consensus.

13 These topics may be taught nonetheless. However, they do not appear in the curricula, which may indicate that they are not emphasized.

14 Our findings also raise a larger question about the structure of undergraduate psychology programs in the UK. These are generally 3-year programs (or 4-year programs in Scotland) in which students are expected to learn quantitative and qualitative skills and conduct a “substantial piece of research [that] typically involves the collection of original empirical data from participants” (British Psychological Society, 2019). Meanwhile, the following themes emerged from our analysis: (a) finite teaching capacity and (b) student-capacity limits, coupled with (c) the need to improve foundational skills and (d) a tension between quantitative and qualitative methods. Some other countries (e.g., United States, Canada) generally offer 4-year psychology programs offering specialization as either a bachelor’s of science or a bachelor’s of arts while allowing students to select from a range of optional modules and requiring substantial final-year projects only from students in honors programs. The results from our present study brought our attention to these differences, although this study does not (and was not designed to) shed light on the benefits and drawbacks of program structures in different countries.

15 We also removed items that reached consensus in Round 1, which made it impossible for an item to go from consensus to nonconsensus. We did this to ensure Round 2 did not take too long to complete (in Round 2, participants also saw a graph for each item). We are not concerned that many items not presented in Round 2 would have lost consensus because most of these items had high percentages of essential ratings, ratings were generally stable across rounds, and all ratings with a percentage of essential ratings above 55% in Round 1 increased this percentage in Round 2 (except one item that had a 1% decrease).
