# Supplementary material for: A Delphi Study to Strengthen Research-Methods Training in Undergraduate Psychology Programs
Source: Adv Methods Pract Psychol Sci. Author manuscript; Available in PMC 2025 Mar 18. (PMC7617501; doi:10.1177/25152459231213808)
Supplement: Supplementary material [file EMS203643-supplement-Supplementary_material.docx]

**SUPPLEMENTARY MATERIAL**

Preprint available at: <https://doi.org/10.31234/osf.io/gp9aj>

Manuscript accepted for publication in *Advances in Methods and Practices in Psychological Science* on 24 Oct 2023

**A Delphi study to strengthen research methods training in undergraduate psychology programmes**

Robert T. Thibault^1,2†^ (
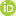
0000-0002-6561-3962)

Deborah Bailey-Rodriguez^3^ (
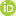
 0000-0002-9931-563X)

James E. Bartlett^4^ (
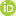
 0000-0002-4191-5245)

Paul Blazey^5^ (
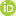
 0000-0002-8149-9514)

Robin J. Green^6^ (
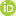
 0000-0002-2615-4409)

Madeleine Pownall^7^ (
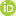
 0000-0002-3734-8006)

Marcus R. Munafò^2,8^ (
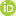
[0000-0002-4049-993X)](https://orcid.org/0000-0002-4049-993X)

^1^Meta-Research Innovation Center at Stanford (METRICS), Stanford University, ^2^School of Psychological Science, University of Bristol, ^3^Psychology Department, Faculty of Science & Technology, Middlesex University, UK, ^4^School of Psychology and Neuroscience, University of Glasgow, UK, ^5^Faculty of Medicine, Dept of Family Practice, University of British Columbia, ^6^School of Psychology and Vision Sciences, University of Leicester, ^7^School of Psychology, University of Leeds, UK, ^8^MRC Integrative Epidemiology Unit at the University of Bristol

^†^Correspondence should be addressed to Robert T. Thibault; E-mail: [robert.thibault@stanford.edu](mailto:robert.thibault@stanford.edu)

24 October 2023

**Supplementary Material A. Details regarding the involvement of the BPS in this Delphi study.**

This supplement provides a detailed outline of how the BPS was involved in this Delphi study.

**Planning.** In March 2022, the project lead (Robert Thibault) contacted the BPS to share the results from a study his group previously conducted on the content of quantitative education in undergraduate psychology education (TARG Meta-Research Group, 2022), and to propose running a consensus study. In May 2022, Thibault and Marcus Munafò presented their previous research and proposal for Undergraduate Programme Directors at the BPS Programme Liaison Day meeting. At this time, the BPS Partnership and Accreditation Committee was already planning to update their accreditation standards relatively soon. They update these standards every 5 years or so. The BPS Accreditation Operations Manager (Patricia Lyons) was our main point of contact from the BPS.

**Design.** Together, Thibault and Lyons assembled a Steering Committee for this Delphi study. The Steering Committee, led by Thibault, then developed the questionnaire and study protocol. They did so with guidance from a colleague specialising in consensus processes (Paul Blazey), and in line with suggestions from Lyons. Thibault and Lyons communicated regularly via email as the study was being designed. After seeking permission to do so, Lyons approved the survey before it was opened to participants. We also shared the survey and study protocol with the Chair of the BPS Undergraduate Education Committee (Simon Goodson) before releasing it, but did not explicitly request his approval. Goodson was also the chair of the Advisory Group for the 2023 QAA Benchmark Statement Review for Psychology.

**Advertising.** The BPS helped advertise the study by sending an email invitation to participate to their mailing lists for Student Ambassadors, Programme Directors, and Psychological Professions Network.

**Analysis.** The analyses and writing of the paper were performed independently of the BPS, except for that one Steering Committee member for this Delphi study (Robin Green) was also the co-lead of the Research Methods Working Group part of the BPS Undergraduate Education Committee.

**Dissemination.** Thibault presented the recommendations to the BPS Undergraduate Education Committee (who are responsible for developing the accreditation standards) in July 2023. Steering committee members Deborah Bailey-Rodriguez and James Bartlett presented the results for the BPS Research Board in October 2023.

**Implementation.** The decisions for how to implement our recommendations are left to the BPS. The updated research methods standards are first drafted by the Research Methods Working Group part of the BPS Undergraduate Educations Committee (on which Steering Committee member Robin Green is also a member). They do so by combining our recommendations with other evidence (e.g., reviewing textbooks and pedagogical literature, consulting with stakeholders). The updated standards are then reviewed and further updated by the Undergraduate Education Committee and then implemented by the Partnerships and Accreditation Team. Throughout this process, our team is available to respond to any questions they may have about the Delphi study and our recommendations.

**Supplementary Material B. Survey home page (incl. literature review and consent form)**

[Participants viewed the text below before starting Round 1 of the Delphi study.]

Welcome to the **Survey on Research Methods Education in Undergraduate Psychology Programmes**, run in partnership with the British Psychological Society.

Before beginning the survey, please take a moment to read the following sections of this page:

1. Study summary
2. Stakeholder groups
3. Literature review
4. Consent form / participant information sheet
5. **Study Summary**

This study asks members of the UK psychology community about the research methods skills they believe psychology undergraduates should learn. The British Psychological Society (BPS) has partnered with us to run this study and they plan to use the results to inform the upcoming version of their accreditation standards.

This is a two-round survey (a Delphi study). The first round is similar to a normal survey. However, you will be able to comment on each question and suggest additional questions. Approximately a week after the first round closes, you will be invited to participate in the second round. In round 2, you will be shown the responses of other participants who completed round 1 and will be asked to re-rate some questions. A few questions will be added or modified based on comments from round 1.

**2. Stakeholder groups**

On the next page, you will be asked to select which stakeholder group you are part of (according to the instructions below):

A. Student in psychology (or recent graduate)

B. Research methods instructor in psychology

C. Academic based in psychology

D. Non-academic working in psychology

If you are an undergraduate or graduate student in psychology, please select option ‘A’ regardless of whether you teach or do research. If you are no longer a student, do not fit into any of the other 3 stakeholder groups, and completed your undergraduate degree less than 3 years ago, select option ‘A’.

If you teach or coordinate psychology research methods in an undergraduate or Masters conversion programme, and are not a student, please select option ‘B’, regardless of whether you are also an academic or non-academic psychologist.

If you are both an academic and non-academic psychologist (e.g., work for industry and a university), select the group that you feel represents you best.

**If you do not fit into one of these four stakeholder groups, you are ineligible to participate in this survey—in this case, please do not proceed.**

**3. Literature review**

**Why run this study?**

To help research methods education keep abreast with advances in research practice and education. The results will inform the upcoming British Psychological Society’s ‘Standards for the accreditation of undergraduate, conversion and integrated Masters programmes in psychology’*.*

This review provides a quick summary on research methods education in UK undergraduate psychology programmes. More detailed reports are available, [here](https://s3.eu-west-2.amazonaws.com/assets.creode.advancehe-document-manager/documents/hea/private/resources/tt_maths_psychology_1568037242.pdf), [here](https://online.ucpress.edu/collabra/article/8/1/38037/193269/Statistics-Education-in-Undergraduate-Psychology-A), and [here](http://clok.uclan.ac.uk/24942/1/Gibson%20Sullivan%20changing%20culture%20aam.pdf). A list of relevant articles appears in the references at the bottom of this page.

There are no correct or incorrect answers to this survey. This summary is to help orient you as a survey participant. Please provide answers based on your own thoughts.

**Statistics in psychology research**

Statistical shortcomings and errors are common in psychology research^e.g.,[1](https://www.zotero.org/google-docs/?broken=Nkfkyd)^. They can increase the prevalence of biased results and lead to a distorted and uncertain evidence base^e.g.,[2](https://www.zotero.org/google-docs/?broken=8RpZNg)^. A 2015 British Academy report[^3^](https://www.zotero.org/google-docs/?broken=AHn4iK) stated that *“*A co-ordinated and continuous effort at improving quantitative skills across all phases of education and employment…is therefore now urgently needed.”

**Quantitative research methods education**

Only a few articles describe the quantitative skills taught in undergraduate psychology programmes. They find minimal changes to the content of undergraduate statistics modules in the United States over 20 recent years[^4,5^](https://www.zotero.org/google-docs/?broken=r3AEOx) and that the emphasis psychology programmes place on null hypothesis significance testing appears out of step with modern statistical thought in the psychological sciences (e.g., estimation, uncertainty, and open science)[^5–8^](https://www.zotero.org/google-docs/?broken=BL5HxK).

Open Science practices are not yet widespread across psychology education. Nonetheless, relevant teaching resources exist^e.g.,[9,10](https://www.zotero.org/google-docs/?broken=XmuxJf)^ and incoming students support Open Science norms[^11^](https://www.zotero.org/google-docs/?broken=wMTuNe)**.**

As of 2019, few UK undergraduate psychology programmes have publicly available syllabi for their quantitative research methods modules.[^8^](https://www.zotero.org/google-docs/?broken=dRcsPx)

**Software and resources**

Of 27 UK undergraduate psychology programmes assessed in 2019, 26 use SPSS, 8 use Excel, and 1 to 3 use R, depending on whether option modules are included.[^8^](https://www.zotero.org/google-docs/?broken=qjdHpL) Andy Field’s *Discovering Statistics using IBM SPSS Statistics*[^12^](https://www.zotero.org/google-docs/?broken=48jaWk) was the most commonly used textbook. Field has written an equivalent book for R[^13^](https://www.zotero.org/google-docs/?broken=RoitjN) and open source online textbooks exist (e.g., [PsyTeachR](https://psyteachr.github.io/)).

**Qualitative research methods education**

Although qualitative research skills have been part of the BPS accreditation standards since 2004, there is limited research on how they are taught.[^14,15^](https://www.zotero.org/google-docs/?broken=hfCSzi) Data suggests that some instructors and students see qualitative research as inferior to quantitative research[^14,16^](https://www.zotero.org/google-docs/?broken=8yutqQ) and that additional training and resources are needed to effectively teach qualitative methods.[^15,17^](https://www.zotero.org/google-docs/?broken=aCGPwA)

There is currently no established consensus on how qualitative approaches are taught in UK psychology programmes. This can present difficulties, but also allows for instructors to teach to their strengths.[^18^](https://www.zotero.org/google-docs/?broken=8m7gk7)

**Teaching and evaluation formats**

Of 27 UK psychology programmes, many research methods modules mentioned dedicating hours to workshop, labs, and practicals. However, none mentioned teaching formats such as a flipped classroom, resequenced content, or collaborative learning. While free online modules gain popularity (e.g., [Improving your statistical inferences](https://www.coursera.org/learn/statistical-inferences)) it remains unclear whether undergraduate instructors are leveraging these resources.

Research on quantitative education suggests that using a problem solving framework and collaborative learning environment can reduce anxiety around statistics and improve learning outcomes.[^19,20^](https://www.zotero.org/google-docs/?broken=wL1b5C) Both instructors and students identify statistics anxiety as a key factor inhibiting their development of quantitative skills.[^21^](https://www.zotero.org/google-docs/?broken=Drje5g) Several modules, however, grade 100% based on exams[^8^](https://www.zotero.org/google-docs/?broken=gNbiL3), which can induce anxiety and fail to mimic the contexts in which students will use quantitative skills in their future.

**Policies and guidelines**

The [BPS provides accreditation standards for undergraduate programmes](https://cms.bps.org.uk/sites/default/files/2022-07/Undergraduate%20Accreditation%20Handbook%202019.pdf), which includes a page on research methods. They also provide a [24-page supplementary guidance](https://web.archive.org/web/20200801055239/https:/www.bps.org.uk/sites/www.bps.org.uk/files/Accreditation/Research%20Methods%20-%20Undergraduate%20Programmes%20WEB.pdf) for teaching research methods. The national bodies in other countries we checked (e.g., United States, Canada) do not accredit psychology programmes.

The accreditation standards consist of broad statements such as “students should be able to analyse, present and evaluate quantitative and qualitative data”. It is difficult to assess whether such standards are being met.

The present study aims to provide more detailed and clear-cut information to help update the BPS accreditation standards.

**References**

[1. Nuijten, M. B., Hartgerink, C. H. J., van Assen, M. A. L. M., Epskamp, S. & Wicherts, J. M. The prevalence of statistical reporting errors in psychology (1985–2013). *Behavior Research Methods* **48**, 1205–1226 (2016).](https://www.zotero.org/google-docs/?broken=VxiY3D)

[2. Simmons, J. P., Nelson, L. D. & Simonsohn, U. False-positive psychology: Undisclosed flexibility in data collection and analysis allows presenting anything as significant. *Psychological Science* **22**, 1359–1366 (2011).](https://www.zotero.org/google-docs/?broken=JuRs7M)

[3. The British Academy. *COUNT US IN QUANTITATIVE SKILLS FOR A NEW GENERATION*. https://www.thebritishacademy.ac.uk/documents/220/Count-Us-In.pdf (2015).](https://www.zotero.org/google-docs/?broken=KqmQ4W)

[4. Friedrich, J., Buday, E. & Kerr, D. Statistical training in psychology: A national survey and commentary on undergraduate programs. *Teaching of Psychology* **27**, 248–257 (2000).](https://www.zotero.org/google-docs/?broken=jNxqmX)

[5. Friedrich, J., Childress, J. & Cheng, D. Replicating a national survey on statistical training in undergraduate psychology programs: Are there “New statistics” in the new millennium? *Teaching of Psychology* **45**, 312–323 (2018).](https://www.zotero.org/google-docs/?broken=kr9se2)

[6. Calin-Jageman, R. J. & Cumming, G. The New Statistics for Better Science: Ask How Much, How Uncertain, and What Else Is Known. *The American Statistician* **73**, 271–280 (2019).](https://www.zotero.org/google-docs/?broken=TKSU5O)

[7. Anglin, S. M. & Edlund, J. E. Perceived need for reform in field-wide methods and the teaching of replication, interpretation, and transparency. *Psychology Learning and Teaching* **19**, 60–76 (2020).](https://www.zotero.org/google-docs/?broken=pkZ7Aw)

[8. TARG Meta-Research Group. *Statistics education in undergraduate psychology: A survey of UK curricula*. https://osf.io/jv8x3 (2022) doi:10.31234/osf.io/jv8x3.](https://www.zotero.org/google-docs/?broken=Rr2fmb)

[9. Pownall, M. *et al.* Embedding open and reproducible science into teaching: A bank of lesson plans and resources. *Scholarship of Teaching and Learning in Psychology* No Pagination Specified-No Pagination Specified (2021) doi:10.1037/stl0000307.](https://www.zotero.org/google-docs/?broken=QJ0gng)

[10. Pennington, C. *A Student’s Guide to Open Science: Using the Replication Crisis to Reform Psychology*. (Open University Press, 2023).](https://www.zotero.org/google-docs/?broken=v6IkTc)

[11. Beaudry, J. L., Williams, M., Philipp, M. C. & Kothe, E. What do incoming university students believe about open science practices in psychology? Preprint at https://doi.org/10.31234/osf.io/rq3zs (2021).](https://www.zotero.org/google-docs/?broken=8DmNgy)

[12. Field, A. Discovering statistics using IBM SPSS statistics. in *Statistics* (2013).](https://www.zotero.org/google-docs/?broken=Ebig1n)

[13. Field, A., Miles, J. & Field, Z. *Discovering Statistics Using R*. (SAGE Publications Ltd, 2012).](https://www.zotero.org/google-docs/?broken=JA6sRw)

[14. Gibson, S. & Sullivan, C. A changing culture? Qualitative methods teaching in U.K. psychology. *Qualitative Psychology* **5**, 197–206 (2018).](https://www.zotero.org/google-docs/?broken=qsDd01)

[15. Hugh-Jones, S., Madill, A., Gibson, S., Keane, K. & Beestin, L. A national survey of qualitative research methods teaching in UK HE psychology departments. (2012).](https://www.zotero.org/google-docs/?broken=2029U0)

[16. Clarke, V. & Braun, V. *Methods: Teaching thematic analysis*. https://www.bps.org.uk/volume-26/edition-2/methods-teaching-thematic-analysis (2013).](https://www.zotero.org/google-docs/?broken=DdeLVI)

[17. Wiggins, S., Gordon-Finlayson, A., Becker, S. & Sullivan, C. Qualitative undergraduate project supervision in psychology: current practices and support needs of supervisors across North East England and Scotland. *Qualitative Research in Psychology* **13**, 1–19 (2016).](https://www.zotero.org/google-docs/?broken=MKZupM)

[18. Gibson, S. & Sullivan, C. Teaching Qualitative Research Methods in Psychology: An Introduction to the Special Issue. *Psychology Learning & Teaching* **11**, 1–5 (2012).](https://www.zotero.org/google-docs/?broken=ne4n3L)

[19. Spiegelhalter, D. *The Art of Statistics: How to Learn from Data*. (2019).](https://www.zotero.org/google-docs/?broken=EAvrFE)

[20. Research Articles. https://www.youcubed.org/evidence/research-articles/.](https://www.zotero.org/google-docs/?broken=RG1bRv)

[21. Field, A. P. *Skills in Mathematics and Statistics in Psychology and tackling transition*. https://s3.eu-west-2.amazonaws.com/assets.creode.advancehe-document-manager/documents/hea/private/resources/tt_maths_psychology_1568037242.pdf (2014).](https://www.zotero.org/google-docs/?broken=SXmui5)

**4. Consent form / Participant information sheet**

Thank you for your interest in completing this anonymous Delphi survey. Please take time to read the following information carefully and discuss it with others if you wish. Your participation is voluntary.

**What is the purpose of the research?**

We are interested in learning what the psychology community in the UK thinks that undergraduate psychology students should learn in terms of research methods. More specifically, we are running this study in partnership with the British Psychological Society (BPS) and they plan to use the results to inform their upcoming accreditation standards for undergraduate psychology education. We are inviting any psychologist, psychology instructor, or psychology student in the UK, or with a link to the UK psychology community, to participate.

**How much time will the study take?**

This is a two-round survey. The first round should take approximately 20-30 minutes, depending on whether you provide comments on the questions. It will be open for two weeks, and you can save your progress and return to the survey anytime in these two weeks.

The second round will begin approximately one week after the first round is completed. It should take approximately 20-30 minutes, will be open for two weeks, and progress can be saved.

**What will happen to the results of this questionnaire?**

When the project is finished, we will share the data with the British Psychological Society. We will analyse the data and report the findings. We may report this in a scientific journal and/or present at a scientific meeting. If you would like a copy of the final paper, you may request this by contacting robert.thibault@bristol.ac.uk. Data will be treated in the following ways:

**What will happen to my data?**

Your involvement in the study will remain confidential. This information will only be available to research staff and national bodies which monitor whether research studies are conducted properly. Your study data will be anonymised. This means that it will be given an identification number and any identifying information about you will be removed.

(1) Answers to multiple-choice questions will be stored as open data on the Open Science Framework (osf.io) and the University of Bristol Research Data Repository. They will not include identifying information. Open data are made available, free of charge, to anyone interested in the project, or who wishes to conduct their own analyses of the data.

(2) Comments and open-ended questions will also be stored as open data on  the Open Science Framework and the University of Bristol Research Data Repository. Parts of responses may be redacted to maintain anonymity. Although these data are anonymous and will contain redactions of information that may compromise anonymity, depending on the content of your response, they may still be identifiable.

**Why open data?**

Open access to research findings and access to data is considered best research practice and is a requirement of many funding bodies and journals. As a large proportion of research is publicly funded, the outcomes of the research should be made publicly available. Sharing data helps to maximise the impact of investment through wider use, and encourages new avenues of research.

**Can I withdraw my data after I have taken part?**

Yes, before the data are made open. Although the study team will not be able to identify which data are yours, the DelphiManager team will be able to associate a unique identifier with your email address and your responses. This information is necessary for a Delphi study because respondents need to be reminded of their round 1 responses, when responding to round 2. To withdraw your data, email the study lead at [robert.thibault@brsitol.ac.uk](mailto:robert.thibault@brsitol.ac.uk). After the study is complete and the data are made open, you will no longer be able to withdraw your data.

**Who has reviewed/approved this work?**

This project has received ethics approval from the University’s Faculty of Life Sciences Research Ethics Committee at the University of Bristol [ENTER ID WHEN ACCEPTED]. If you have any concerns related to this project, please direct them to the Psychological Science Human Research Ethics Committee, via Liam McKervey (liam.mckervey@bristol.ac.uk or +44 (0)117 928 7841).

**Contact details**

 If you have any questions about the study, please contact Robert Thibault (robert.thibault@bristol.ac.uk) at the School of Psychological Science, University of Bristol.

I understand that after the study the data will be made “open data”. I understand that this means the anonymised data will be publicly available and may be used for purposes not related to this study, and it is unlikely that someone will be able to identify me from these data.

By clicking the “Register” button below, you are providing your informed consent to participate in this study.

[Participants viewed the text below before starting Round 1 of the Delphi study.]

## **Questions - Round 2**

Please rate the question below.

The figure presents the distribution of responses to this question from each stakeholder group in Round 1. The figures are presented to provide you with information about the perspectives of each stakeholder group.

This information may or may not impact your rating. Please note that the barcharts are presented as percentages and each group had a different number of respondents. The column "X" in each barchart depicts the percentage of respondents who selected "Unable to rate".

Respondents in the Research Methods Instructor group, may also be academic or non-academic psychologists. However, their responses are only displayed in the Research Methods Instructor figure. Respondents in the Academic Psychologist and Non-Academic Psychologist groups, are not research methods instructors. To see the full definition for each stakeholder group, click on the 'About' tab in the bottom left corner of the page on scroll to section 2. Stakeholder Groups.

**Supplementary material C. Deviations from the preregistered protocol**

- Whereas the protocol stated “All participants who completed Round 1 will be invited via email to participate in Round 2”, the manuscript states **“**All participants who began Round 1 were invited via email to participate in Round 2.” We made this decision after Round 1 was complete. The goal was to increase the number of respondents in Round 2, even if they didn’t complete Round 1. Nonetheless, the final dataset only includes participants who completed both rounds. We used data from participants who didn’t complete either Round 1 or Round 2 for a sensitivity analysis.
- The protocol states “We will advertise this Delphi study via mailing lists, psychology related websites, and social media (e.g., Twitter, Mastodon, LinkedIn)”. In the end, we were not successful in getting psychology organisations to post our study on their website. Our study team did not personally advertise the study on Mastodon or LinkedIn, although others may have.
- The protocol did not specify how we planned to analyse the textual data. This was decided after data collection was complete.
- The protocol did not specify how we would summarise the items reaching consensus or how we would go from the results to our recommendations. These decisions were made after viewing the data. The results informed these processes.
- The protocol states “We will also perform a sensitivity analysis by excluding participants who failed the attention check question.” Instead, we decided to exclude these participants from the main analysis and then perform a sensitivity analysis with these participants. This decision was made before viewing the data.
- We report several instances of quantitative data that were not specifically prespecified, including Figure 2 (flowchart), Table 1 (participant characteristics), Supplementary Table 1, Supplementary Table 2, and Supplementary Table 3.

#

# **Supplementary Material D. ACCORD Checklist (ACcurate COnsensus Reporting Document)** [**https://doi.org/10.1101/2023.08.22.23294261**](https://doi.org/10.1101/2023.08.22.23294261)

*Note, instead of using line numbers, we have inserted (i) the section(s) of the manuscript, (ii) the verbatim text from the manuscript in quotation marks, and/or (iii) a brief explanation of how we address the item.

| **Item number** | **Manuscript section** | **Item wording** | **Help text** | **Reported?**  **(line number)*** |
| --- | --- | --- | --- | --- |
| T1 | Title | Identify the article as reporting a consensus exercise and state the consensus methods used in the title. | For example, Delphi or Nominal Group Technique. | “A Delphi study to strengthen research methods training in undergraduate psychology programmes” |
| I1 | Introduction | Explain why a consensus exercise was chosen over other approaches | n/a | Section 2. Methods. First paragraph. |
| I2 | Introduction | State the aim of the consensus exercise, including its intended audience and geographical scope (national, regional, global). | n/a | Section 1.1 Study objective |
| I3 | Introduction | If the consensus exercise is an update of an existing document, state why an update is needed, and provide the citation for the original document. | n/a | n/a |
| M1 | Methods > Registration | If the study or study protocol was prospectively registered, state the registration platform and provide a link. If the exercise was not registered, this should be stated. | Recommended to include the date of registration. | “We preregistered a protocol before advertising the study (<https://osf.io/5h7bu>). Deviations from the preregistered protocol are outlined in Supplementary Material C.” |
| M2 | Methods > Selection of SC and/or panellists | Describe the role(s) and areas of expertise or experience of those directing the consensus exercise. | For example, whether the project was led by a chair, co-chairs or a steering committee and, if so, how they were chosen. List their names if appropriate, and whether there were any subgroups for individual steps in the process. | Section 2.1 Delphi Steering Committee |
| M3 | Methods > Selection of SC and/or panellists | Explain the criteria for panellist inclusion and the rationale for panellist numbers. State who was responsible for panellist selection. | n/a | Section 2.2 Participants  Supplementary Material A |
| M4 | Methods > Selection of SC and/or panellists | Describe the recruitment process (how panellists were invited to participate). | Include communication/advertisement method(s) and locations, numbers of invitations sent, and whether there was centralised oversight of invitations or if panellists were asked/allowed to suggest other members of the panel. | Section 2.2 Participants  Supplementary Material A  Supplementary Material E |
| M5 | Methods > Selection of SC and/or panellists | Describe the role of any members of the public, patients or carers in the different steps of the study. | n/a | 2.1 Delphi Steering Committee  2.2 Participants  Patients were not involved, as this was not health research. |
| M6 | Methods > Preparatory research | Describe how information was obtained prior to generating items or other materials used during the consensus exercise. | This might include a literature review, interviews, surveys, or another process. | Section 2. Methods. Paragraph 3.  Footnote 2  Supplementary Material B |
| M7 | Methods > Preparatory research | Describe any systematic literature search in detail, including the search strategy and dates of search or the citation if published already. | Provide the details suggested by the reporting guideline PRISMA and the related PRISMA-Search extension. | n/a, a systematic literature search was not performed. |
| M8 | Methods > Preparatory research | Describe how any existing scientific evidence was summarised and if this evidence was provided to the panellists. | n/a | Section 2. Methods. Paragraph 3.  Footnote 2  Supplementary Material B |
| M9 | Methods > Assessing consensus | Describe the methods used and steps taken to gather panellist input and reach consensus (for example, Delphi, RAND-UCLA, nominal group technique). | If modifications were made to the method in its original form, provide a detailed explanation of how the method was adjusted and why this was necessary for the purpose of your consensus-based study. | Section 2.3 Definition of consensus |
| M10 | Methods > Assessing consensus | Describe how each question or statement was presented and the response options. State whether panellists were able to or required to explain their responses, and whether they could propose new items. | Where possible, present the questionnaire or list of statements as supplementary material. | Section 2.4 Survey  Section 2.5 Open-ended questions  Supplementary Material B  Supplementary Material G  Supplementary Material H |
| M11 | Methods > Assessing consensus | State the objective of each consensus step. | A step could be a consensus meeting, a discussion or interview session, or a Delphi round. | Figure 1 outlines the steps. The related text throughout Section 2 Methods details of each step. |
| M12 | Methods > Assessing consensus | State the definition of consensus (for example, number, percentage, or categorical rating, such as ‘agree’ or ‘strongly agree’) and explain the rationale for that definition | n/a | Section 2.3 Definition of consensus |
| M13 | Methods > Assessing consensus | State whether items that met the pre-specified definition of consensus were included in any subsequent voting rounds | n/a | “Questions that reached consensus in Round 1 were removed from Round 2.” |
| M14 | Methods > Assessing consensus | For each step, describe how responses were collected, and whether responses were collected in a group setting or individually. | n/a | 2.4 Survey |
| M15 | Methods > Assessing consensus | Describe how responses were processed and/or synthesised. | Include qualitative analyses of free-text responses (for example, thematic, content or cluster analysis) and/or quantitative analytical methods, if used. | 2.6 Analyses  Open code, available at [https://osf.io/hpsq4](https://osf.io/hpsq4/) |
| M16 | Methods > Assessing consensus | Describe any piloting of the study materials and/or survey instruments. | Include how many individuals piloted the study materials, the rationale for the selection of those individuals, any changes made as a result, and whether their responses were used in the calculation of the final consensus. If no pilot was conducted, this should be stated. | Footnote 6 |
| M17 | Methods > Assessing consensus | If applicable, describe how feedback was provided to panellists at the end of each consensus step or meeting. | State whether feedback was quantitative (for example, approval rates per topic/item) and/or qualitative (for example, comments, or lists of approved items), and whether it was anonymised. | Supplementary Material G. |
| M18 | Methods > Assessing consensus | State whether anonymity was planned in the study design. Explain where and to whom it was applied and what methods were used to guarantee anonymity. | n/a | “We used a modified-Delphi technique to systematically elicit anonymous, asynchronous, and iterative input from a range of stakeholders” |
| M19 | Methods > Assessing consensus | State if Steering Committee was involved in the decisions made by the consensus panel. | For example, whether the steering committee or those managing consensus also had voting rights | “Members of the Steering Committee did not participate in the Delphi.” |
| M20 | Methods > Participation | Describe any incentives used to encourage responses or participation in the consensus process. | For example, were invitations to participate reiterated, or were participants reimbursed for their time | “ No incentives were offered for participation.” |
| M21 | Methods > Participation | Describe any adaptations to make the surveys/meetings more accessible. | For example, the languages in which the surveys/meetings were conducted and whether translations or plain language summaries were available. | We did not adapt any material to make it more accessible. |
| R1 | Results | State when the consensus exercise was conducted. List the date of initiation and the time taken to complete each consensus step, analysis, and any extensions or delays in the analysis. | n/a | **“**Round 1 was open from 8 February 2023 to 17 March 2023. We extended the length of Round 1 due to delays in sending emails to the BPS mailing lists and because we were receiving fewer responses than expected. The initially low response rate may have been caused in part by the University and College Union (UCU) strikes in February and March 2023. Round 2 was open from 6 April 2023 to 28 April 2023.” |
| R2 | Results | Explain any deviations from the study protocol, and why these were necessary. | For example, addition of panel members during the exercise, number of consensus steps, stopping criteria; report the step(s) in which this occurred. | Supplementary Material C |
| R3 | Results | For each step, report quantitative (number of panellists, response rate) and qualitative (relevant socio-demographics) data to describe the participating panellists. | n/a | Figure 2.  Table 1.  Open data with summary statistics (see Data Availability statement). |
| R4 | Results | Report the final outcome of the consensus process as qualitative (for example, aggregated themes from comments) and/or quantitative (for example, summary statistics, score means, medians and/or ranges) data. | n/a | 3.3 Consensus summary results  Table 3.  3.4 Thematic analysis of the open-ended questions |
| R5 | Results | List any items or topics that were modified or removed during the consensus process. Include why and when in the process they were modified or removed. | n/a | “Items that reached consensus in Round 1 were removed from Round 2. No items were modified between Round 1 and Round 2.” |
| D1 | Discussion | Discuss the methodological strengths and limitations of the consensus exercise. | Include factors that may have impacted the decisions (for example response rates, representativeness of the panel, potential for feedback during consensus to bias responses, potential impact of any non-anonymised interactions). | Section 4.4 Limitations |
| D2 | Discussion | Discuss whether the recommendations are consistent with any pre-existing literature and, if not, propose reasons why this process may have arrived at alternative conclusions. | n/a | Section 4.2 Relation to the literature |
| O1 | Other information | List any endorsing organisations involved and their role. | n/a | Section. Endorsing organisations  Supplementary Material A |
| O2 | Other information | State any potential conflicts of interests, including among those directing the consensus study, and panellists. Describe how conflicts of interest were managed. | n/a | “Robin J. Green is a member of the BPS Undergraduate Education Committee, who were tasked with updating the accreditation standards. All other authors declare no competing interests.” |
| O3 | Other information | State any funding received and the role of the funder. | Specify, for example, any funder involvement in the study concept/design, participation in the steering committee, conducting the consensus process, funding of any medical writing support. This could be disclosed in the methods or in the relevant transparency section of the manuscript. Where a funder did not play a role in the process or influence the decisions reached, this should be specified. | “Robert Thibault is supported by a general support grant awarded to METRICS from Arnold Ventures and a postdoctoral fellowship from the Canadian Institutes of Health Research. This project did not receive specific funding. This publication is the work of the authors and Robert Thibault will serve as guarantor for the contents of this paper. The funders have no role in the preparation of this manuscript or the decision to publish.” |

#

# **Supplementary Material E. Participant invitation templates**

We provided organisations interested in advertising our study with the following invitation templates. The decision on the exact invitation text to use will be left to each individual organisation to decide.

**Short version (for social media)**

Have thoughts about what psychology undergrads should learn? Then participate in this Delphi study run in partnership with the British Psychological Society [LINK TO DELPHI]. Your input will shape the future of quantitative and qualitative research methods education in the UK.

**Long version (for email)**

Dear [colleagues],

We would like to invite you to participate in a study about undergraduate psychology education in the UK [LINK TO DELPHI].

This study aims to reach a consensus on the qualitative and quantitative research methods that UK psychology undergraduates should learn. The study is run in partnership with the British Psychological Society (BPS) and they will use the results to inform the upcoming version of their accreditation standards for undergraduate programmes.

This study uses the Delphi technique, which systematically elicits two-rounds of anonymous and asynchronous input from a range of stakeholders. For more details, please follow the link to the study.

We invite instructors, academic psychologists, non-academic psychologists, and psychology students to participate.

If you have any questions or comments, please send them directly to the study lead at robert.thibault@bristol.ac.uk

Kind regards,

[signature]

**Supplementary Material F. Study registration form**


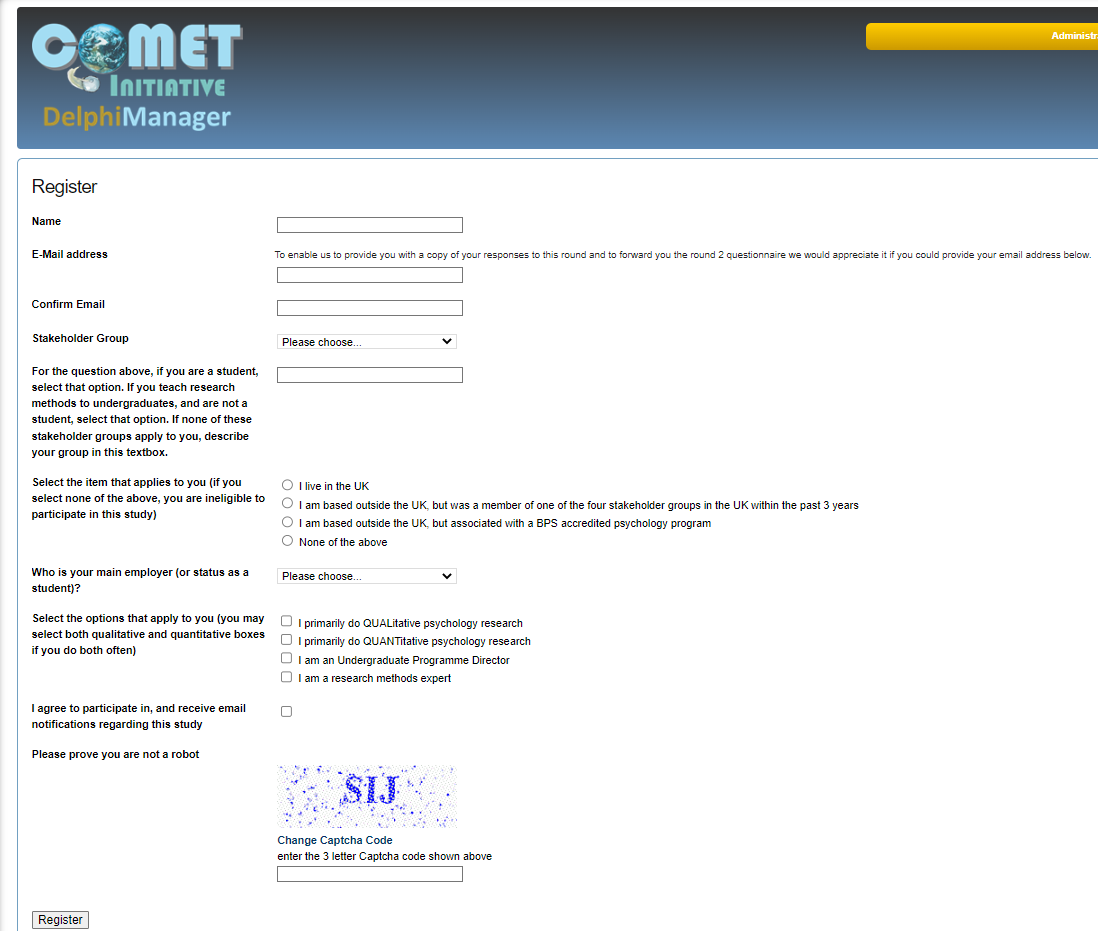


***Drop-down menu 1***


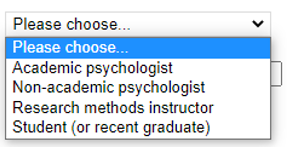


***Drop-down menu 2***

**
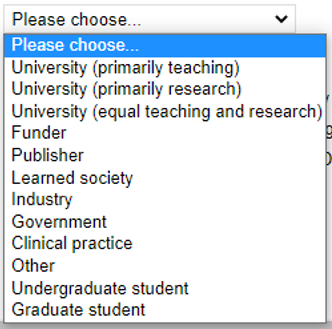
**

**Supplementary Material G. Additional figures and tables**


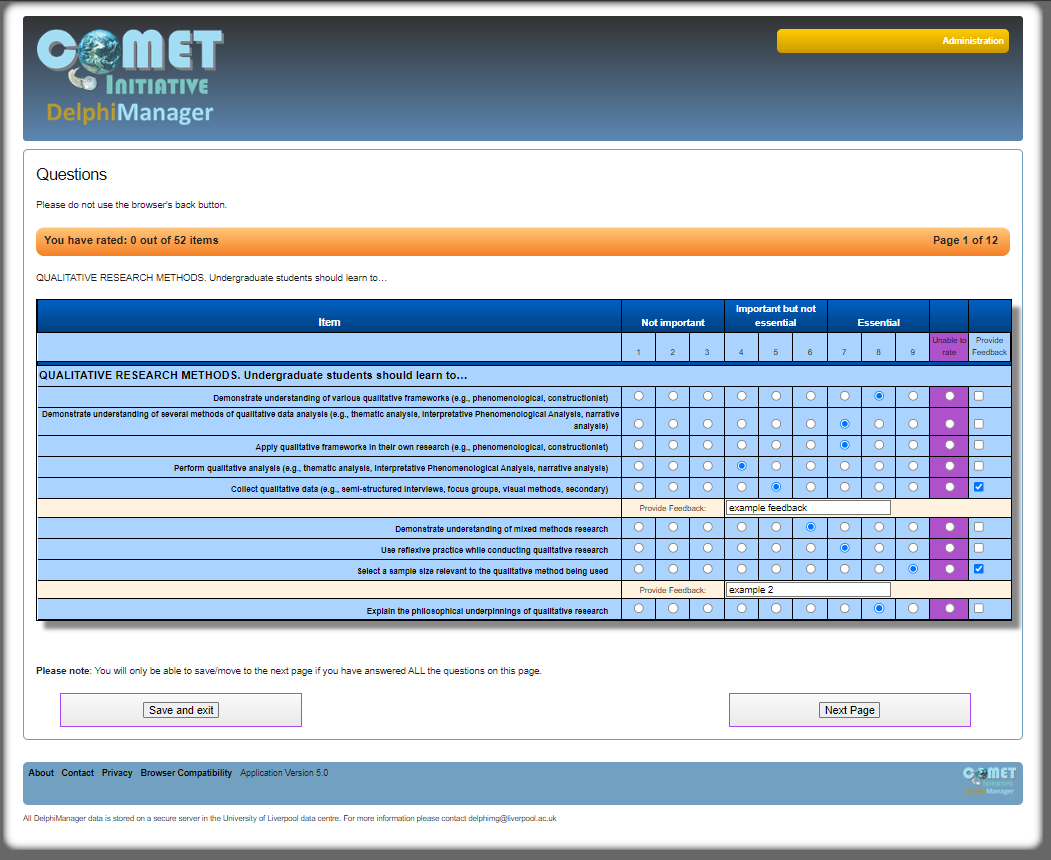


**Supplementary Figure 1. Screenshot of DelphiManager Round 1**. Note, this screenshot was taken after the study was complete, and thus the text “0 out of 52 items” appears, whereas “0 out of 72” appeared for participants.

**
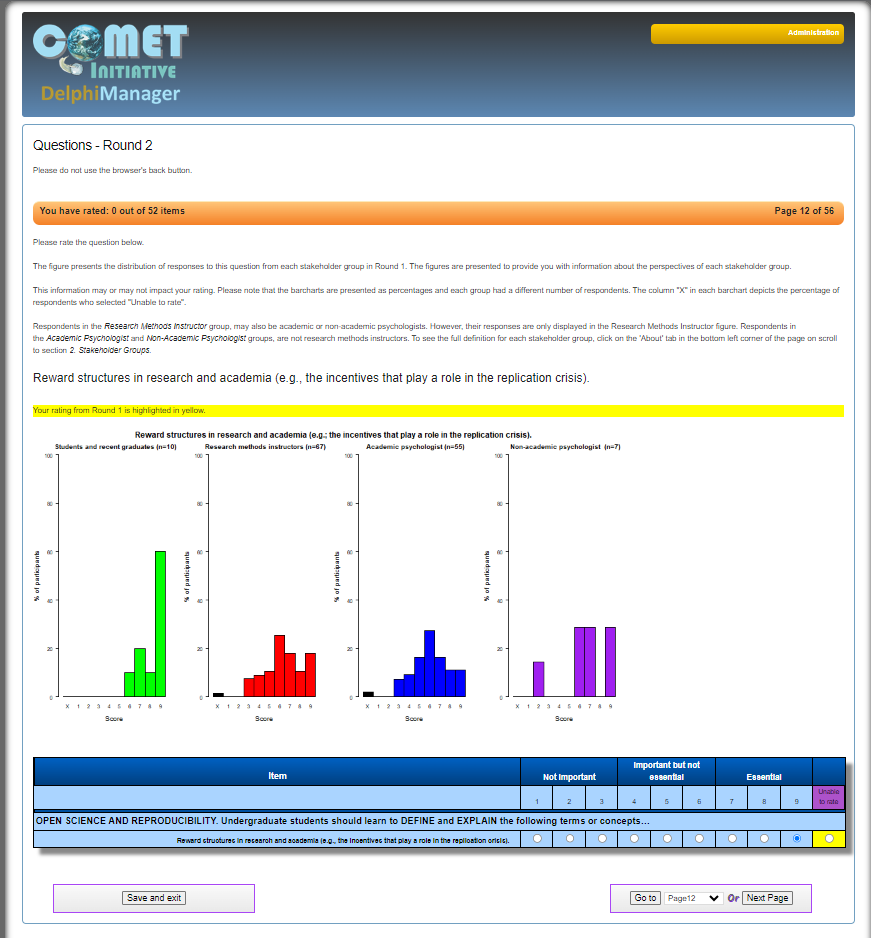
**

**Supplementary Figure 2. Screenshot of DelphiManager Round 2**. In Round 2, items were presented one at a time alongside feedback from participants’ ratings from the previous round.

### **Supplementary Table 1. Counts for open-ended responses.** Few participants accounted for a large proportion of the feedback. For example, participant BPSRE00038 provided 15% (33/222) of all open-ended feedback on specific Delphi items. We considered this uneven distribution of open-ended responses when analysing this data. Participants could only leave a single general feedback comment at the very end of each round. Based on the format in which the DelphiManager outputs data, the user_ids from the column ‘items suggested to include’ cannot be linked to the user_ids in the other columns.

| **Feedback on items** | | **Items suggested to include** | | **Reasoning for changing a rating across a boundary** | |
| --- | --- | --- | --- | --- | --- |
| **user_id** | **frequency** | **user_id** | **frequency** | **user_id** | **frequency** |
| BPSRE00038 | 33 | 22 | 28 | BPSRE00015 | 14 |
| BPSRE00005 | 22 | 5 | 5 | BPSRE00156 | 14 |
| BPSRE00087 | 15 | 17 | 4 | BPSRE00025 | 12 |
| BPSRE00029 | 14 | 9 | 3 | BPSRE00054 | 11 |
| BPSRE00026 | 11 | 10 | 3 | BPSRE00084 | 10 |
| BPSRE00076 | 11 | 11 | 3 | BPSRE00164 | 10 |
| BPSRE00074 | 10 | 12 | 3 | BPSRE00082 | 9 |
| BPSRE00052 | 9 | 13 | 3 | BPSRE00107 | 9 |
| BPSRE00077 | 9 | 23 | 3 | BPSRE00018 | 8 |
| BPSRE00009 | 8 | 2 | 2 | BPSRE00067 | 8 |
| BPSRE00120 | 8 | 3 | 2 | BPSRE00074 | 8 |
| BPSRE00053 | 7 | 16 | 2 | BPSRE00160 | 8 |
| BPSRE00044 | 6 | 20 | 2 | BPSRE00030 | 7 |
| BPSRE00124 | 5 | 21 | 2 | BPSRE00055 | 7 |
| BPSRE00081 | 4 | 28 | 2 | BPSRE00094 | 7 |
| BPSRE00110 | 4 | 1 | 1 | BPSRE00130 | 7 |
| BPSRE00150 | 4 | 4 | 1 | BPSRE00145 | 7 |
| BPSRE00154 | 4 | 6 | 1 | BPSRE00019 | 6 |
| BPSRE00075 | 3 | 7 | 1 | BPSRE00022 | 6 |
| BPSRE00079 | 3 | 8 | 1 | BPSRE00034 | 6 |
| BPSRE00167 | 3 | 14 | 1 | BPSRE00052 | 6 |
| BPSRE00047 | 2 | 15 | 1 | BPSRE00076 | 6 |
| BPSRE00050 | 2 | 18 | 1 | BPSRE00088 | 6 |
| BPSRE00057 | 2 | 19 | 1 | BPSRE00089 | 6 |
| BPSRE00082 | 2 | 24 | 1 | BPSRE00151 | 6 |
| BPSRE00089 | 2 | 25 | 1 | BPSRE00005 | 5 |
| BPSRE00091 | 2 | 26 | 1 | BPSRE00008 | 5 |
| BPSRE00106 | 2 | 27 | 1 | BPSRE00077 | 5 |
| BPSRE00139 | 2 | 29 | 1 | BPSRE00108 | 5 |
| BPSRE00152 | 2 | 30 | 1 | BPSRE00115 | 5 |
| BPSRE00008 | 1 |  |  | BPSRE00137 | 5 |
| BPSRE00025 | 1 |  |  | BPSRE00147 | 5 |
| BPSRE00034 | 1 |  |  | BPSRE00154 | 5 |
| BPSRE00035 | 1 |  |  | BPSRE00172 | 5 |
| BPSRE00042 | 1 |  |  | BPSRE00010 | 4 |
| BPSRE00065 | 1 |  |  | BPSRE00042 | 4 |
| BPSRE00094 | 1 |  |  | BPSRE00091 | 4 |
| BPSRE00098 | 1 |  |  | BPSRE00092 | 4 |
| BPSRE00099 | 1 |  |  | BPSRE00136 | 4 |
| BPSRE00162 | 1 |  |  | BPSRE00011 | 3 |
| BPSRE00166 | 1 |  |  | BPSRE00013 | 3 |
|  |  |  |  | BPSRE00017 | 3 |
|  |  |  |  | BPSRE00024 | 3 |
|  |  |  |  | BPSRE00026 | 3 |
|  |  |  |  | BPSRE00038 | 3 |
|  |  |  |  | BPSRE00127 | 3 |
|  |  |  |  | BPSRE00131 | 3 |
|  |  |  |  | BPSRE00141 | 3 |
|  |  |  |  | BPSRE00142 | 3 |
|  |  |  |  | BPSRE00144 | 3 |
|  |  |  |  | BPSRE00152 | 3 |
|  |  |  |  | BPSRE00016 | 2 |
|  |  |  |  | BPSRE00023 | 2 |
|  |  |  |  | BPSRE00028 | 2 |
|  |  |  |  | BPSRE00057 | 2 |
|  |  |  |  | BPSRE00066 | 2 |
|  |  |  |  | BPSRE00106 | 2 |
|  |  |  |  | BPSRE00117 | 2 |
|  |  |  |  | BPSRE00124 | 2 |
|  |  |  |  | BPSRE00138 | 2 |
|  |  |  |  | BPSRE00162 | 2 |
|  |  |  |  | BPSRE00029 | 1 |
|  |  |  |  | BPSRE00070 | 1 |
|  |  |  |  | BPSRE00073 | 1 |
|  |  |  |  | BPSRE00075 | 1 |
|  |  |  |  | BPSRE00111 | 1 |
|  |  |  |  | BPSRE00118 | 1 |
|  |  |  |  | BPSRE00120 | 1 |
|  |  |  |  | BPSRE00126 | 1 |
|  |  |  |  | BPSRE00133 | 1 |
|  |  |  |  | BPSRE00149 | 1 |
|  |  |  |  | BPSRE00169 | 1 |
|  |  |  |  | BPSRE00180 | 1 |

**Supplementary Table 2.** **Consensus results sorted by the block (domain) in which the item was presented.**

**
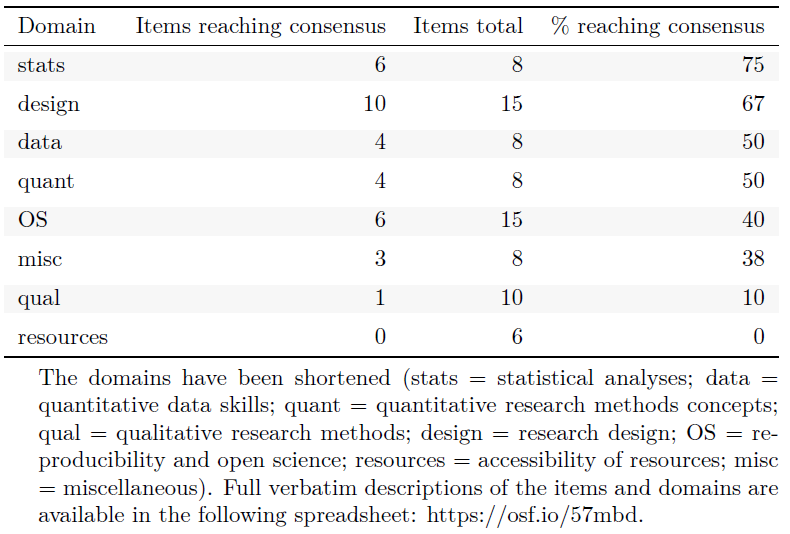
**

**Supplementary Table 3. Sensitivity analyses between Delphi rounds, stakeholder**

**groups, and initial and final samples**

**
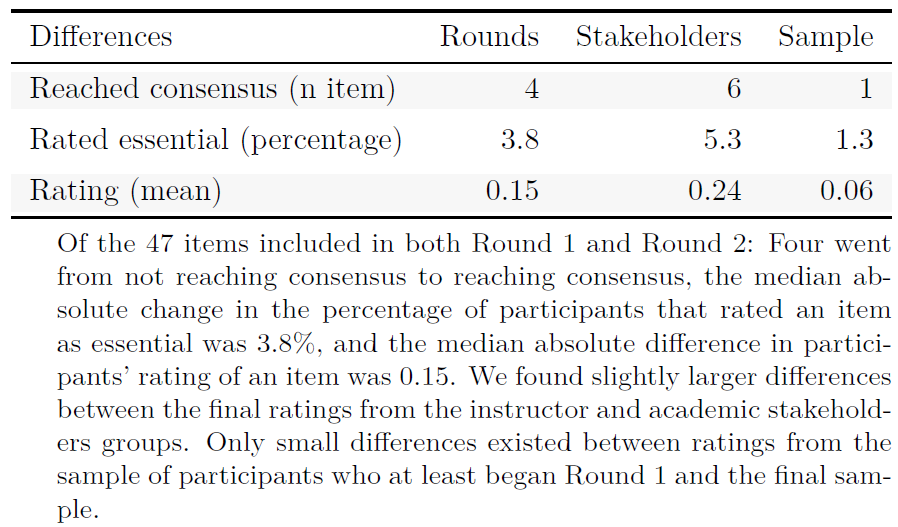
**

**Supplementary Material H. Delphi items**

- Sections were presented in a random order.
- Questions within each section always appeared in the same order (the DelphiManager Software does not allow randomization of the questions within a section).
- Each question…
  - is rated on a scale from 1-9, where 1-3 is 'not important' 4-6 is 'important, but not essential', and 7-9 is 'essential'.
  - has the option to select 'unable to rate'
  - has the option to provide open-ended written feedback

| Number of items to rate in Round 1:  Number of items removed after Round 1:  Number of items added after Round 1:  Number of items in Round 2: | 72  27  7  52 |
| --- | --- |

1. **STATISTICAL ANALYSES.** *Undergraduate students should learn how to calculate/perform…*
   1. Descriptive statistics (e.g., mean, interquartile range)
   2. Significance tests (e.g., t-test, ANOVAs, Pearson’s correlations, chi-squared)
   3. Parameter estimation (e.g., calculating 95% confidence intervals)
   4. Regression
   5. Methods to assess statistical assumptions (e.g., normality)
   6. Effect sizes (e.g., Cohen’s d, odds ratios)
   7. Equivalence testing
   8. Factor analysis
2. **QUANTITATIVE DATA SKILLS.** *Undergraduate students should learn how to…*
   1. Identify and categorise different types of data (e.g., binary, continuous, categorical)
   2. Clean data (e.g., remove bad data, rearrange data—i.e., “wrangle” data)
   3. Represent data visually (e.g., create histograms, line graphs)
   4. Use a programming language to manage and analyse data (e.g., R or Python)
   5. Use a statistical analysis package with a graphical user interface (e.g., SPSS or JASP)
   6. Simulate data
   7. Use descriptive statistics effectively before learning to perform inferential statistical tests
3. **QUANTITATIVE RESEARCH METHODS CONCEPTS.** Undergraduate *students should learn to define and explain the following concepts…*
   1. The existence of different statistical frameworks, including frequentist statistics and Bayesian statistics
   2. The existence of different statistical approaches, including parameter estimation and significance testing
   3. The importance of descriptive statistics and how they differ from inferential statistics
   4. The difference between statistical significance and practical significance (also called clinical, theoretical, or biological significance)
   5. The value of exploratory research and how it differs from confirmatory research
   6. Probability and randomness
   7. Psychometrics (e.g., scale construction)
4. **QUALITATIVE RESEARCH METHODS.** Undergraduate *students should learn to…*
   1. Demonstrate understanding of various qualitative frameworks (e.g., phenomenological, constructionist)
   2. Demonstrate understanding of several methods of qualitative data analysis (e.g., thematic analysis, Interpretative Phenomenological Analysis, narrative analysis)
   3. Apply qualitative frameworks in their own research (e.g., phenomenological, constructionist)
   4. Perform qualitative analysis (e.g., thematic analysis, Interpretative Phenomenological Analysis, narrative analysis)
   5. Collect qualitative data (e.g., semi-structured interviews, focus groups, visual methods, secondary)
   6. Demonstrate understanding of mixed methods research
   7. Use reflexive practice while conducting qualitative research
   8. Critically appraise qualitative research (e.g., using qualitative criteria)
   9. Select a sample size relevant to the qualitative method being used
   10. Explain the philosophical underpinnings of qualitative research
5. **RESEARCH DESIGN.** Undergraduate *students should learn how to…*
   1. Formulate a research question
   2. Explain the difference between a research question and a hypothesis
   3. Design a study to answer a specific research question (including the selection of an appropriate research method and analytic approach)
   4. Create a sampling plan and data collection plan (for both quantitative and qualitative research; and in line with the research question and method of analysis)
   5. Determine a smallest effect size of interest (SESOI) for quantitative research.
   6. Perform sample size calculations for quantitative research (e.g., power calculations, precision calculations)
   7. Attention check: Please select “3” to confirm you are reading these questions.
   8. Assess validity and reliability
   9. Operationalize all elements of a study
   10. Apply experimental and non-experimental research designs
   11. Apply blinding and randomization when conducting an experiment
   12. Follow accepted reporting guidelines
   13. Design a survey
   14. Identify and assess ethical issues (in both qualitative and quantitative research)
6. **OPEN SCIENCE AND REPRODUCIBILITY.** Undergraduate *students should learn to define and explain the following terms or concepts……*
   1. The “replication crisis”
   2. Philosophy of science
   3. Questionable Research Practices (QRPs) (e.g., selective reporting, p-hacking)
   4. Research misconduct (i.e., fabrication, falsification, and plagiarism)
   5. Replication studies and reproducibility
   6. Generalisability and robustness
   7. Reward structures in research and academia (e.g., the incentives that play a role in the replication crisis).
   8. Preregistration and Registered Reports
   9. Sources of bias (in both qualitative and quantitative research. E.g., sampling bias)
   10. Cognitive biases (and how these drive the replication crisis. E.g., confirmation bias)
   11. Data, code, and material sharing (e.g., open data).
   12. The publication process (including Open Access and peer review)
   13. Meta-research / meta-science (e.g., how these methods shed light on the replication crisis)
   14. Multiverse analyses / many-analyst approaches
   15. Systematic reviews and meta-analysis
7. **ACCESSIBILITY OF RESOURCES.** *Research methods modules in undergraduate psychology should…*
   1. Only use freely available software (e.g., R or JASP, rather than SPSS)
   2. Make their syllabi publicly available (e.g., on the Open Science Framework—OSF)
   3. Provide students with syllabi that include a week-by-week outline of the module contents
   4. Never be entirely graded with closed-book exams
   5. Have a higher staff to student ratio than for non-research methods modules (this could include teaching assistants).
8. **MISCELLANEOUS QUESTIONS**
   1. Students should preregister the quantitative aspects of their final year project.
   2. Students should be allowed to perform a replication as their final year research project
   3. Students should be allowed to conduct their final year project in a team.
   4. Students should have the option to conduct a qualitative, quantitative, or mixed-methods project in their final year research.
   5. Research methods instructors should be given time and support to improve their skills (e.g., to improve their understanding of qualitative methods or learn R, if they plan to teach these skills)
   6. Research methods modules should actively employ teaching and grading methods known to reduce “statistics anxiety”
9. **ITEMS ADDED TO ROUND 2**
   1. Undergraduate students should learn how to: anonymize data
   2. Undergraduate students should learn how to: Consider diverse perspectives when designing a study (e.g.; global approaches; marginalised or vulnerable communities; decolonising methodologies)
   3. Undergraduate students should learn how to: Demonstrate general computer skills for research (e.g.; file structure; version control; spreadsheets; and word processing)
   4. Undergraduate students should learn how to: Identify basic study designs (e.g.; randomized trial; cross-sectional; qualitative designs)
   5. Undergraduate Students should learn how to: Search and collate published research (e.g.; by using databases such as Scopus and reference managers such as Endnote or Zotero)
   6. Undergraduate students should learn to DEFINE and EXPLAIN the following concepts: Alternative measures of effect sizes (e.g.; probability of superiority; Cohen’s U3; number needed to treat)
   7. Research methods MODULES in undergraduate psychology should: Emphasize skills that transfer beyond an academic research context (e.g.; training for the job market; creating an informed citizenry)

[The remainder of the questions were open-ended text responses]

1. **ADDITIONAL OUTCOMES**
   1. If you feel that this survey did not include certain questions you would deem important, please enter them here and provide your ratings. We will review these questions and may include some in the next round of this Delphi study.
2. **COMMENTS**
   1. Please provide any thoughts you have about this Delphi study here.
3. **RATING CHANGE QUESTION** (Round 2 only)
   1. Some of the ratings you have changed have moved across the rating categories eg. from Not Important to Important but not critical. Please could you give a reason for these significant changes:

**Supplementary Material I. Recommendation to the BPS**

We sent a synopsis of this Delphi study and a list of recommendations to the BPS Undergraduate Education Committee on 03 July 2023. This pdf document is available at (https://osf.io/4vd37). The content of this pdf is also provided on the next 3 pages.

**Recommendations for updating the research methods section of the BPS undergraduate accreditation standards**

**Authors.** Robert T. Thibault, Deborah Bailey-Rodriguez, James Bartlett, Paul Blazey, Robin J. Green, Madeleine Pownall, Marcus R. Munafò. Correspondence to: robert.thibault@stanford.edu

**Executive summary**

Our team of researchers ran a consensus process. Our goal was to identify the research methods skills that the UK psychology community deems essential for undergraduates to learn. Of 78 items included in the consensus process, 34 reached consensus. We also performed a qualitative analysis of 707 open-ended text responses. Based on our findings, we developed nine Core Recommendations for updating the research methods section of the [BPS accreditation standards](https://cms.bps.org.uk/sites/default/files/2022-07/Undergraduate%20Accreditation%20Handbook%202019.pdf). These include emphasising data skills, research design, descriptive statistics, critical analysis, qualitative methods, and both significance testing and parameter estimation; as well as giving precedence to foundational skills, promoting transferable skills, and creating space within curricula to enable these recommendations. A full account of the consensus process, including methods, results, and interpretation is available here [temporary link to manuscript draft removed].

**Methodology**

We used the Delphi technique, which systematically elicits anonymous, asynchronous, and iterative input from a range of stakeholders. With input from the BPS Accreditation Operations Manager (Patricia Lyons) and Chair of the BPS Undergraduate Education Committee (Simon Goodson), a steering committee of 5 researchers and instructors developed a survey that consisted of 78 items for participants to rate as ‘not important’, ‘important, but not essential’, or ‘essential’ for undergraduate students in UK psychology programmes to learn. Invitations to participate were extended via email and social media. Anyone from the UK psychology community was welcome to participate. 103 research methods instructors, academic psychologists, non-academic psychologists, and students completed the consensus process. As is common in Delphi studies, we considered consensus reached if at least 75% of participants in each stakeholder group rated an item as ‘essential’. Participants had several opportunities to provide open-ended feedback and we assessed the stability of responses with a second survey round. Similar consensus processes have been used to develop standards in over 200 medical education programmes [(Humphrey-Murto et al., 2017)](https://doi.org/10.1097/acm.0000000000001812).

**Core Recommendations**

We developed these recommendations based on ratings to the 78 Delphi items and participants’ open-ended feedback. Each recommendation is presented as a general concept that the BPS could integrate into their accreditation standards, as well as specific text that could be added as a bullet point to *Section 2.1.4.g Research Methods*. Following the format of the current accreditations standards, the bullet points below are preceded by the text “Students should be able to…”.

1. **Require a strong understanding of data and quantitative data skills.**
   - “Identify and categorise different types of quantitative data (e.g., categorical, continuous), clean and wrangle data, and represent data visually (e.g., histograms, line graphs)”.
2. **Emphasise general skills in research design.**
   - Edit the item “generate and explore hypotheses and research questions drawing on relevant theory and research” to “formulate and operationalise research questions and hypotheses, drawing on relevant theory and research; and explain the difference between a research question and hypothesis”.
   - “Identify basic study designs, differentiate exploratory and confirmatory research, and apply experimental and non-experimental research designs”.
3. **Prioritise a solid foundation in descriptive statistics.**
   - “Calculate descriptive statistics (e.g.; mean; interquartile range), and explain how descriptive statistics differ from inferential statistics”
4. **Provide students with a framework to critically assess research claims.**
   - “Identify and explain sources of bias (e.g., sampling bias), cognitive biases (e.g., confirmation bias), questionable research practices (e.g., selective reporting), generalisability and robustness, research misconduct, replication studies and reproducibility”.
5. **Raise the prominence of qualitative methods throughout the accreditation standards.**
   - “Understand several qualitative methods, perform qualitative analyses, present qualitative research, and critically appraise qualitative research (e.g. using qualitative criteria).”
6. **For inferential statistics, require that parameter estimation techniques, such as confidence intervals and effect sizes, be taught alongside significance testing.**
   - “Understand both parameter estimation and significance testing, calculate effect sizes and confidence intervals, and perform regressions”.
   - “Explain the difference between statistical significance and practical significance”.
7. **Give precedence to teaching foundational research methods skills (as outlined in Recommendations 1-5).**
   - Proficiently apply foundational qualitative and quantitative skills (e.g., descriptive statistics) before learning more advanced techniques (e.g., inferential statistics)”.
8. **Promote content that elucidates how research methods skills transfer beyond academia.**
   - *Section 2.2 Teaching and learning* “Education providers must deliver content that demonstrates how students can apply research methods skills and psychological literacy in diverse real-world situations, as well as academic and non-academic employment pathways.
9. **Enable Recommendations 1-8 by encouraging research methods education throughout the programme, focusing on fewer skills in greater depth, and offering optional modules for more advanced research methods skills.**
   - Change the statement: “Research methods must be delivered at Level 5 or Level 6” to “Research methods should be delivered throughout Levels 4-6 and can be integrated into core area modules”.
   - Edit the statement “carry out empirical studies involving a variety of methods of data collection, including experiments, observation, questionnaires, interviews and focus groups” to “carry out at least one quantitative and one qualitative study, which may include…”
   - Edit the statement “use a variety of psychological tools, including specialist software, laboratory equipment and psychometric instruments” to “identify a variety of psychological tools, including…and use at least some of these”.

**Elaboration**

Ratings were very high and consensus reached for data skills, basic research design, descriptive statistics, and inferential statistics. Almost 90% of participants rated it essential that students learn to use descriptive statistics effectively before learning to perform inferential statistics. Our qualitative analysis also highlighted the need for students to master foundational quantitative and qualitative skills, rather than attempt to perform analyses that they do not understand. These findings challenge the null-hypothesis significance testing (NHST) centric approach taken in many research methods curricula. The BPS can take a leadership role by de-emphasising NHST in favour of robust foundational quantitative and qualitative skills. Students must understand what analyses they are performing and why they are doing them.

Critical assessment is a pillar throughout the 2019 accreditation standards, but specific concepts and tools are not outlined. By updating the standards to include specific items, such as learning about replication and sources of bias, the BPS can champion a more structured approach to critically assess the psychology literature and other research claims.

Open-ended comments suggested that students should learn how to answer a research question and focus on fewer technical abilities. With this in mind, research methods education could adopt a problem-solving approach by teaching students how to ask a clear question, design an effective research plan, identify what data is needed to answer their question, and select an appropriate analysis plan or statistical procedure to answer their question. The BPS accreditation standards could outline this problem-solving approach as an alternative to statistics-centric methods curricula.

**Additional considerations**

44 items did not reach consensus. These items spanned topics including module format, final-year projects, computer skills, approaches to research, and advanced analysis techniques. Many of these items received a high-level of agreement, but fell short of consensus. There was **not** consensus *against* teaching these items. Notably, the survey was also designed with a quantitative focus.

Delphi studies are not designed to identify the best educational content or teaching methods. Instead, our results represent topics which a broad sample of the academic psychology community in the UK believe are essential for undergraduates to learn. This study measured community norms and expectations.
